# Nano-guided cell networks as conveyors of molecular communication

**DOI:** 10.1038/ncomms9500

**Published:** 2015-10-12

**Authors:** Jessica L. Terrell, Hsuan-Chen Wu, Chen-Yu Tsao, Nathan B. Barber, Matthew D. Servinsky, Gregory F. Payne, William E. Bentley

**Affiliations:** 1 Fischell Department of Bioengineering, University of Maryland, 2330 Jeong H. Kim Engineering Building, College Park, Maryland 20742, USA; 2 Institute for Bioscience and Biotechnology Research, University of Maryland, College Park, Maryland 20742, USA; 3 U.S. Army Research Laboratory, 2800 Powder Mill Road, Adelphi, Maryland 20783, USA

## Abstract

Advances in nanotechnology have provided unprecedented physical means to sample molecular space. Living cells provide additional capability in that they identify molecules within complex environments and actuate function. We have merged cells with nanotechnology for an integrated molecular processing network. Here we show that an engineered cell consortium autonomously generates feedback to chemical cues. Moreover, abiotic components are readily assembled onto cells, enabling amplified and ‘binned' responses. Specifically, engineered cell populations are triggered by a quorum sensing (QS) signal molecule, autoinducer-2, to express surface-displayed fusions consisting of a fluorescent marker and an affinity peptide. The latter provides means for attaching magnetic nanoparticles to fluorescently activated subpopulations for coalescence into colour-indexed output. The resultant nano-guided cell network assesses QS activity and conveys molecular information as a ‘bio-litmus' in a manner read by simple optical means.

## Abstract (web summary)

Living cells have to interpret and react to changes in local environmental conditions. Here the authors exploit that by combining magnetic nanoparticles and bacterial quorum sensing to investigate and convey alterations in the molecular landscape.

## Graphical abstract

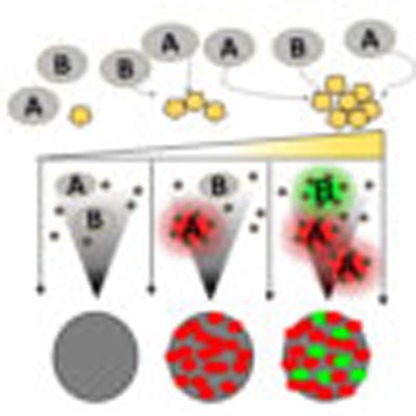

It has become increasingly apparent that a wealth of molecular information exists, which, when appropriately accessed, can provide feedback on biological systems, their componentry and their function. Thus, there is a developing niche that transcends length scales to concurrently recognize molecular detail and at the same time provide understanding of the overall system^1^,^2^. An emerging scheme is to develop nano- to micro-scaled tools that intimately engage with biological systems through monitoring and interacting at the molecular level, with synthetic biology being one such tool^3^,^4^,^5^,^6^,^7^.

While synthetic biology is often viewed as an innovative means for ‘green' product synthesis through the genetic rearrangement of cells, their biosynthetic capabilities and their regulatory networks can instead be tuned for executive function^8^,^9^,^10^. That is, cells can be rewired to survey molecular space^3^,^11^,^12^ as they have sophisticated capabilities to recognize, amplify and transduce chemical information^13^. Further, they provide a means to connect biological systems with traditional microelectronic devices and in doing so present a potential interface between chemically based biomolecular processing and conventional vectors of information flow, such as electrons and photons^14^,^15^,^16^. Specifically, through engineered design, cell-based molecular processing can be further coupled to enable external abiotic responses. Cells, then, represent a versatile means for mediating the molecular ‘signatures' common in complex environments, or in other words, they are conveyors of molecular communication^17^,^18^,^19^.

Further, beyond clonal cell-based sensors, there is an emerging concept of population engineering to establish microorganisms in deliberate networks that enable enriched system identification through a combination of distinctive yet coexistent behaviours, including, perhaps, competitive or cooperative features^8^,^20^,^21^,^22^,^23^,^24^,^25^. We posit the use of cell populations assembled in parallel¸ where multiple microbes with distinct molecular recognition capabilities work congruently. An advantage is that populations, as opposed to few cells, can facilitate thorough sampling since the presence of many cells increases their spatial breadth and per-cell data contributions (Fig. 1a). Each cellular unit undergoes independent decision-making and contributes a datum to its entire constituency. The prevalence of data provided within the population, then, substantiates a collective output by the system based on the molecular landscape. As follows in a multi-population system, molecular input thus influences the outcomes of each population, and elicits plural responses when the molecular input ranges overlap the ranges of the sensing populations^21^, which can define classification boundaries (Fig. 1a). Cell-mediated classification was posited *in silico* by Didovyk *et al*.^21^, where reporter libraries with randomized sensitivities to a molecular cue elicit concentration-dependent fluorescent patterns and these are elucidated by population screening . In the present construct, multiple populations enable multiplexed analysis, resulting, here, in a response gradation that is designed to index the molecular input ‘signature'. Consequently, the fed-back information becomes transfigured beyond a dose-dependent cell-by-cell analysis. That is, the output is predicated by the comparison between the populations rather than accumulation of response within a total population.

With population engineering as a premise for enriched molecular information processing, we engineered cell species, each to achieve an appropriate output through genetic means. There is conceptual basis for incorporation into networks, such as through mobile surveillance and position-based information relay^26^,^27^. Hence, it is conceivable that, in addition to autonomous molecular recognition and processing afforded by synthetic biology, the use of physical stimuli to enable cell response could confer similar networking properties^28^,^29^. For example, the complete information-processing ‘repertoire' can be expanded beyond specific cell responses by the integration of external stimuli that serve to collate cell populations^30^. Specifically, we envision integration of nanomaterials that enable co-responses to molecular inputs, such that cell populations employ traditional reporting functions, that is, fluorescence marker expression, as well as responses that enable additional processing via the integration of stimuli-responsive abiotic materials (Fig. 1b).

In our example, cells are engineered to respond by permitting the attachment of magnetic nanoparticles (mNPs), such that each fluorescent cell becomes receptive to a magnetic field. Thus, the combination of cell-nanoparticle structures provides further dimensionality for the conveyance of molecular information (via magnetic stimulation). That is, without magnetic collation the fully distributed system would harbour diffuse responses; a magnetically stimulated system results in acute output due to a filtering and focusing effect (Fig. 1b)^31^,^32^, allowing binned information to be readily, and fluorescently, conveyed.

The detection and interpretation of signalling molecules in our example is based on a microbial communication process known as quorum sensing (QS). The molecules, autoinducers (AIs), are secreted and perceived within a microbial community; once accumulated, the AI level indicates that the population size has reached a ‘quorum'^33^,^34^. By surpassing a threshold concentration, the AI signalling coordinates population-wide phenotypic changes^35^. We have designed a QS information processor that utilizes two cell populations to independently interrogate natural microbial communities and generate information about QS activity by accessing AI-2 (ref. 36). Each cell population becomes ‘activated' in response to a characteristic AI-2 level by expressing a fluorescent marker and a streptavidin-binding peptide (SBP) on the outer membrane^38^. SBP provides a means for collating data by binding mNPs that are introduced into the community. Using a post-processing magnetic sweep, the system as a whole interprets a molecular landscape and refines output into colour-categorized, or ‘binned,' states (no fluorescence, red, or red and green) through (1) parallel population processing and (2) acute focusing (Fig. 1c).

The use of engineered cells as data-acquiring units and selectively equipping each with functional nanomaterials to form a redistributable processing system merges two paradigms: decentralized, active probing at a molecular scale and self-organization of units through structured dependencies on stimuli^42^. The population-based system overall contributes categorized feedback about a biological environment.

## Results

### Surface expression of SBP and fluorescent protein fusions

**Figure 2:**
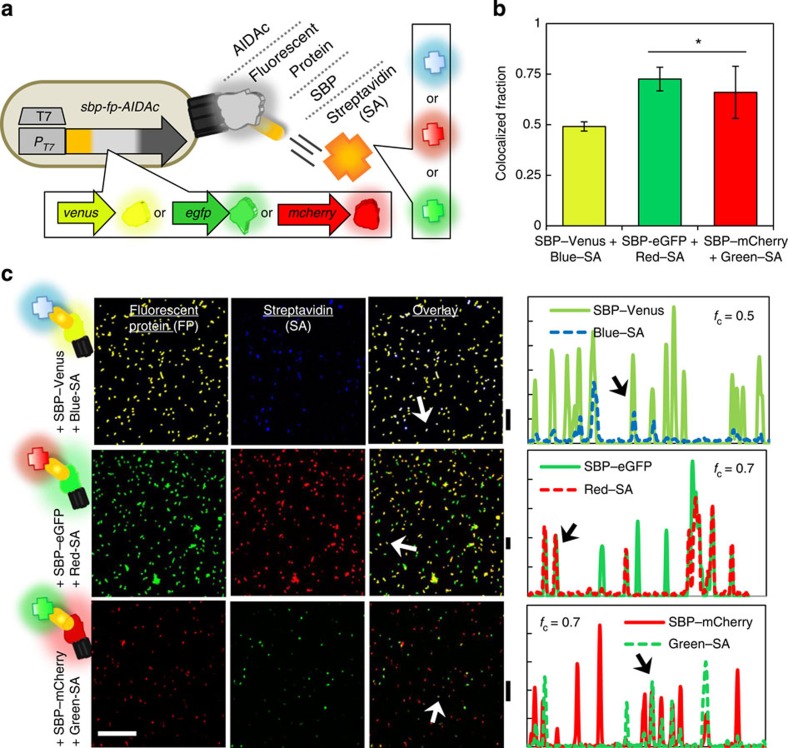
Cells express functional, interchangeable protein components indicating both fluorescence and ability for streptavidin-linked surface coupling. (**a**) A T7 cassette was used to express chimeric proteins consisting of a membrane autotransporter domain (AIDAc), one of several fluorescent proteins and a streptavidin-binding peptide (SBP). Fluorophore-tagged streptavidin (SA) was used to bind SBP. (**b**) Of cells expressing fluorescent proteins (FP), those also marked by SBP coupling are represented as a ‘colocalized fraction (*f*_c_),' plotted with image analysis-based s.d. of at least five replicates. The asterisk ‘*' denotes *f*_c_ that +SBP–eGFP and+SBP–mCherry are statistically equivalent (*f*_c_∼0.7) by *t*-test and greater than +SBP–Venus cells. (**c**) Composite images show cell fluorescence (Column I) from the fluorescent protein (FP); labelled streptavidin using orthogonal filter sets (Column II); and an overlay of both (Column III). Arrows indicate representative cells with strong colocalization. Plotted in Column IV are the fluorescence mean grey values (*y*-axis) from a representative horizontal slice of the composite image (*x*-axis). Vertical bars displayed between Columns III and IV identify the position of each analysed slice. Arrows indicate peaks that match the highlighted cells in Column III. *f*_c_ values are noted. Fluorophores with non-overlapping spectra were paired. Row 1, Venus expression (yellow-green) was paired with Dylight405-labelled SA (blue). Row 2, eGFP expression (green) was paired with Alexafluor594-labeled SA (red). Row 3, mCherry expression (red) was paired with Alexafluor488-labeled SA (green). Scale bar in lower left, 50 μm.

First, we established expression of a fusion protein consisting of a fluorescent marker (enhanced green fluorescent protein (eGFP) and variants) and SBP. Importantly, for SBP to function as a coupling agent between cells and mNPs, we used AIDAc (kindly shared by J. Larssen)^40^ to export the chimeric protein to *Escherichia coli's* outer surface. Translocation to a cell's surface utilizes a signal peptide (for inner membrane translocation) and AIDAc as an outer membrane autotransporter pore^38^,^39^,^40^,^41^, with the passenger protein linked to each. In Fig. 2a, we depict expression of three different constructs using Venus, eGFP and mCherry for optical transmission, and the AIDAc translocator domain for surface localization. These constructs are mapped in Supplementary Fig. 1. After induction with isopropyl B-D-1-thiogalactopyranoside (IPTG), cultures were probed for surface expression of the SBP portion of the tagged fluorescent protein. Cells were incubated with fluorescently labelled streptavidin; the fluorophore of the streptavidin probe was orthogonal to the expressed fluorescent protein. The multiple fluorescence emissions were analysed by confocal microscopy without spectral overlap. The fraction of cells (*f*_c_) that exhibit colocalized fluorescent protein and the fluorescently-labeled streptavidin is reported in Fig. 2b, showing that SBP–Venus cells bound streptavidin at a slightly lower frequency than SBP–mCherry and SBP–eGFP, which exhibited statistically similar fractions (*f*_c_=0.7).

That is, microscopy results related to the colocalization analysis are depicted for pairings of Venus and blue-streptavidin (SA), eGFP and red-SA, and mCherry and green-SA (Fig. 2c). Strong signals were observed in both filter sets (the fluorescent protein (Column I) and the labelled streptavidin (Column II)). Overlaying each image reveals colocalization, as indicated in Column III, where arrows point to examples of strong colocalization. In addition, Column IV plots fluorescence intensities across horizontal sections of the images, where cells that exhibit colocalized fluorescence are indicated by superimposed peaks. For +pSBP–Venus cells, those with both a blue and yellow signal are observed as pale blue–violet in the overlaid image. Cells with +pSBP–eGFP and +pSBP–mCherry and labelled streptavidin emit both green and red signals; their colocalization appears yellow. Controls shown in Supplementary Fig. 2, verify that fluorescent streptavidin (all colours) has specificity for only SBP-expressing cells over negative controls. Colocalization indicates that not only are both components of the fusion, SBP and the fluorescent protein, expressed, but that SBP is accessible to bind streptavidin on the cell's surface. This is the first use of AIDAc for cell surface anchoring of fluorescent proteins, each having been functionalized with an affinity peptide.

### Cell hybridization via mNPs

**Figure 3:**
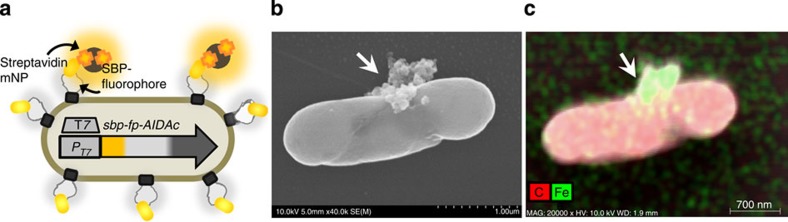
Cells equipped with magnetic nanoparticles (mNPs) via streptavidin-mediated interaction with surface-expressed proteins. (**a**) Cell surface binding of streptavidin-conjugated magnetic nanoparticles occurs via surface-anchored streptavidin-binding peptide (SBP). The fusion of T7-expressed SBP-fluorescent protein (FP)-AIDAc enables the cell surface accessibility. (**b**) Scanning electron micrograph of an *E. coli* cell with surface-bound particles. (**c**) Element map of carbon (red) and iron (green) through energy-dispersive spectroscopy.

Given that expression of a fluorescent protein tagged with SBP enabled external binding of streptavidin, we employed this interaction for fastening streptavidin-functionalized materials directly to the cell surface. We chose streptavidin-conjugated mNPs, 100 nm in diameter (an order of magnitude smaller than a cell), for binding to a cell surface (Fig. 3a) to impart the abiotic magnetic properties. Scanning electron microscopy (SEM) was used to observe surface interaction between cell surface-expressing SBP and streptavidin-functionalized mNPs. Supplementary Fig. 3a,b shows electron micrographs of *E. coli* cells (dimensions 1.5–2 μm in length) and the mNPs (∼100 nm in diameter). The SEM image in Fig. 3b, shows a magnetically isolated SBP-expressing cell with streptavidin-mNPs. The sample was prepared by mixing SBP-expressing cells with streptavidin-mNPs, then collecting or ‘focusing' into a magnetized pellet via magnetic field, then separating from unbound cells in the supernatant. The cells were then washed and resuspended. In Fig. 3b, clusters of surface-bound mNPs are observed. In addition, the elemental composition was analysed with energy-dispersive X-ray spectroscopy, shown in Fig. 3c by an element map superimposed with carbon (red) and iron (green). While the cell appears to be of a uniform carbon composition, the particles localized at the cell surface (highlighted with arrows) were found having a strong iron composition; thus, elemental analysis confirmed particle identity as iron oxide mNPs. Additional characterization of magnetic functionality, including detailed SEM and fluorescent microscopic analysis prior to and after application of magnetic fields, is described in the Supplementary Information (Supplementary Fig. 3).

In sum, the well-known affinity interaction between streptavidin and the peptide SBP is harnessed to endow cells with non-natural abiotic properties. Here coupling a functionalized nanomaterial to the surface-displayed peptide physically extends the fusion protein and also adds physical (magnetic) functionality to the cell.

### Linking expression to AI-2 recognition

The expression system for pSBP–Venus was then put under AI-2 control so that the protein is expressed in the presence of AI-2 instead of IPTG. That is, we coupled the native QS signal transduction circuitry to the reporter cassette. To ensure ample expression (as the native operon is fairly weak), we placed expression of T7 RNA polymerase under control of the natural QS circuitry^43^. Phosphorylated AI-2 activates the system through derepression of the regulator LsrR, naturally upregulating AI-2 import and phosphorylation^44^, and, by design, the T7 RNA polymerase on a sensor plasmid^43^. When *sbp–Venus* is included downstream of a T7 promoter region on a second plasmid, expression is then triggered by AI-2 uptake (Supplementary Fig. 4a). Then, we used two host sensor strains engineered to provide varied AI-2 sensitivity (denoted responders ‘A' and ‘B'). In ‘A', *lsrFG*, genes required for internally phosphorylated AI-2 degradation^45^,^46^ are deleted. Also, both strains lack the terminal AI-2 synthase, *luxS*, so they cannot produce AI-2 and, instead, must ‘receive' AI-2 from an external source (Supplementary Fig. 4a). The phenotypic difference between A and B is the threshold level of AI-2 that activates the genetic response^47^,^48^. Fully constructed, these cells are designed to take up and process AI-2 to generate fluorescence output (that co-functions with streptavidin binding).

**Figure 4:**
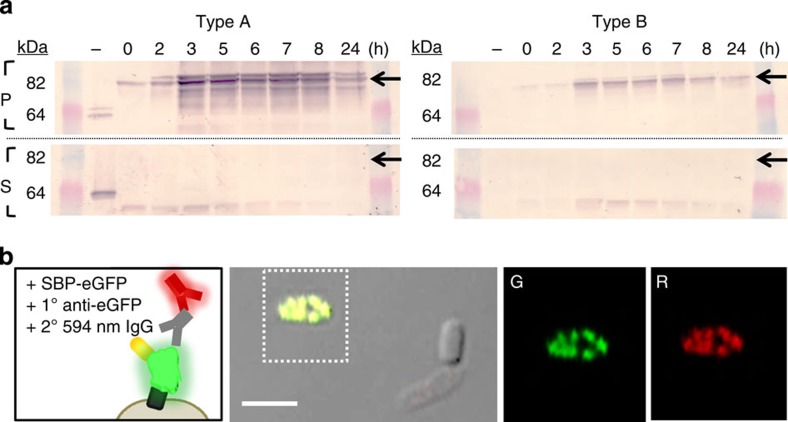
Affinity-based probing for functional analysis of AI-2-induced protein expression. (**a**) 64–82 kDa region of western blot for pelleted (P) and supernatant (S) protein fractions isolated from Type A and B cells. Alkaline phosphatase-conjugated streptavidin was used to target AIDAc–Venus–SBP at expression timepoints. Arrows indicate the expected position of the full fusion protein. (**b**) Immunostaining for assessment of the fluorescent protein surface accessibility. The external surfaces of cells expressing AIDAc–eGFP–SBP were probed with an anti–eGFP and Alexafluor594-labelled antibody pair. A representative overlaid fluorescence and phase contrast image is shown along with fluorescence images of the green (G) and red (R) filters for the boxed-in region. Scale bar, 2 μM.

We next evaluated the kinetics of surface-fusion protein expression and effects on cell growth. The AI-2-induced expression for AIDAc-linked and SBP-tagged fluorescent proteins did not alter growth kinetics for either cell type (Supplementary Fig. 4b,c). Expression efficacy was also evaluated via immunoassay of the outer membrane, probing for AI-2-induced surface display. After induction with 20 μM AI-2, extracts from cell types A and B were size-separated and blotted using alkaline phosphatase-conjugated streptavidin to probe for the SBP-tagged protein fusion (Supplementary Fig. 5). The 88 kDa AIDAc–Venus–SBP protein was only found in the membrane-containing pellet fraction (Fig. 4a). Analogously, protein orientation was assessed by immunolabeling the fluorescent protein. Cell type B transformed with pSBP–eGFP was induced with 20 μM AI-2 overnight; cell surfaces were then probed for eGFP using a mouse anti-GFP primary antibody and red-labelled secondary anti-mouse IgG. Simultaneously, cells were observed using phase contrast and fluorescence confocal microscopy. We noted a punctate pattern for eGFP, which was in one-to-one correspondence with red immunostaining of the surface-expressed protein. The positive staining of eGFP-expressing cells for red fluorescence, contrasted by the absence of negative control immunostaining indicated surface exposure of the fusion (Supplementary Fig. 6). Confocal microscopy confirmed precise colocalization of the eGFP and red-labelled antibodies within the confines of individual cells (Fig. 4b). Therefore, efficient transport of this functionality to the membrane under AI-2 induction was demonstrated in each host.

### Establishing molecular ranges for cell interrogation

**Figure 5:**
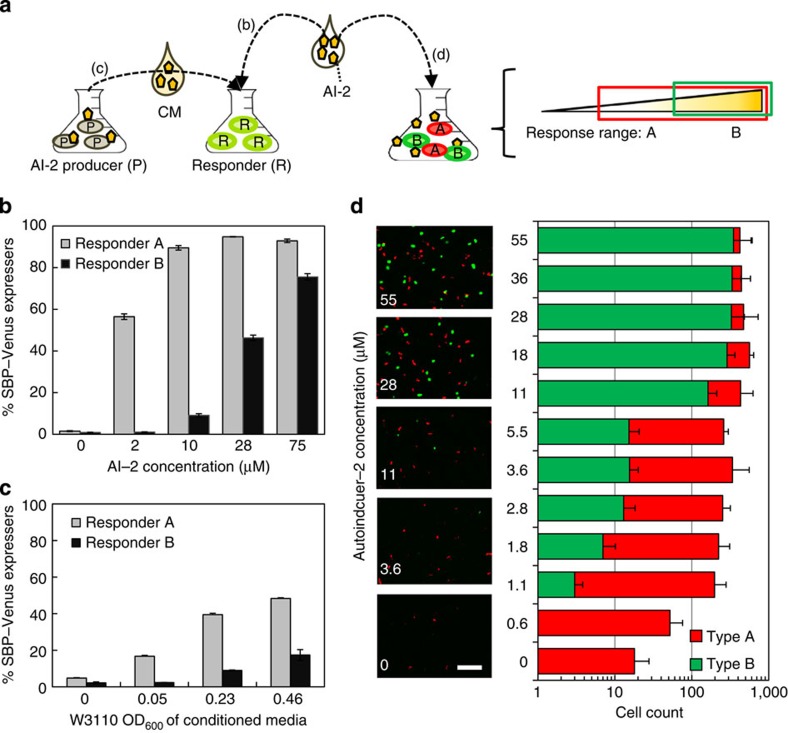
Single and multi-population cell responses to autoinducer-2. (**a**) Fluorescence output is linked to small molecule input, derived from purified or crude sources. Fluorescence from Responders A and B was analysed after exposure to autoinducer-2 (AI-2) in mono and mixed culture environments. (**b**) Venus expression from *in vitro*-synthesized AI-2 added to monocultures of A and B. (**c**) Venus expression from conditioned media (CM) added to monocultures of A and B. CM was isolated from WT W3110 *E. coli* cultures sampled at indicated OD. Data are averages from triplicate cultures with s.d. indicated. (**d**) Red and green fluorescence responses to AI-2 during co-incubation of Responders A (pSBP–mCherry+, red) and B (pSBP–eGFP+, green). Representative fluorescence images show colocalization of red and green cells. Scale bar, 10 μm. The average cell count per responder cell is plotted against AI-2 concentration, as determined by image analysis in quadruplicate. All data are plotted as averages of at least triplicate samples with s.d.

Importantly, the engineered cells each provide a characteristic response to the level of AI-2. Recently, we showed that AI-2 level influences the quorum size of responding engineered populations but does not alter the expression level within each quorum^47^. Here we evaluated our engineered AI-2 responders, again for quorum size (or in other words, percentage of AI-2-responsive cells in the population), this time varying the compositions of molecular input and the configuration of responders (Fig. 5a). First, we added AI-2, synthesized *in vitro*, to each of the two responder populations (Fig. 5b). We also added conditioned medium (CM), the spent medium from an AI-2 producer culture containing metabolic byproducts, as well as AI-2 (refs 36, 49; Fig. 5c). We also mixed the responder populations and added AI-2 to gauge responses in complex cultures (Fig. 5d).

Specifically, in Fig. 5b, A and B populations were incubated at mid-exponential phase with *in vitro*-synthesized AI-2 (refs 50, 51) at concentrations: 0, 2, 10, 28 and 75 μM. After 12 h, samples were observed for fluorescence by confocal microscopy and then quantified by fluorescence-activated cell sorting (FACS; Supplementary Fig. 4c). We found that SBP–Venus expression for responder A cells occurred at the lowest tested level (2 μM AI-2), where 56% of the population expressed SBP–Venus and this fraction increased with AI-2 reaching a maximum of 90% at 28 μM. For type B, a more gradual trend was found; only ∼1% was fluorescent from 0-2 μM, and this increased from 9 to 46% as AI-2 was increased to 28 μM. Finally, the highest fraction of fluorescing cells was found at the highest concentration tested, 75 μM.

We next isolated CM, which contains a dynamic composition of unfiltered metabolites and media components, from W3110 *E. coli* cultures at intervals during their exponential growth, throughout which AI-2 accumulates (AI-2 levels for the samples are indicated in Supplementary Fig. 7). CM aliquots were mixed with either A or B cells and cultured in triplicate for 12 h. Through FACS analysis it was found, again, that a larger subpopulation of A expressed Venus compared with population B at any concentration (Fig. 5c). Statistically relevant expression from B was not apparent until incubated with CM from cultures at an optical density (OD) of 0.23. In all cases, population A recognized AI-2 presence, including from media isolated at a W3110 OD of 0.05, the minimum cell density tested in this study.

The sensitivities of both strains to AI-2-mediated induction corroborate previous literature^10^,^47^. These trends demonstrate that strains engineered for altered sensitivity to molecular cues provide discrimination of concentration level. That is, the identical plasmid expression system was transformed into different hosts, providing robust and distinct levels of expression.

Having developed cell types A and B with differential ability to detect AI-2, we next altered the reporters so that each cell type expressed a unique SBP-fluorescence fusion for colour-coded designation. Cell type A was engineered with pSBP–mCherry and type B with pSBP–eGFP, resulting in red and green fluorescence, respectively. These populations were mixed together in equal proportion at mid-exponential phase, introduced to a range of AI-2 concentrations, and incubated overnight. Populations A and B exhibited equal growth rates when cultured alone and together (Supplementary Fig. 8c); it followed that the cocultures should comprise a 1:1 ratio of each constituent. Fluorescence output is shown by representative images in Fig. 5d. Also in Fig. 5d, the green and red cell count is plotted from a quadruplicate analysis for each input concentration.

Coculturing enables parallel processing as the molecule-rich environment is perceived by each cell, and is processed uniquely per cell type. Yet, since each sensing mechanism is a living and proliferating population, we tested whether the potentially altered dynamics of coculturing would permit the same sensitivities as isolated culturing. We evaluated the Monod-type saturation constant for each population independently and in cocultures. We found, in Fig. 5d, the general trends in response to an increasing AI-2 level were as predicted by modelled response curves (Supplementary Table 4), which were also well-correlated to Fig. 5b data (Supplementary Fig. 8a,b). That is, the saturation constants that describe dependence on AI-2 were unchanged when measured in cocultures. Phenomenologically, as expected, an initial accumulation of red type A responders was found. Then, at higher AI-2 levels, we found an emergence of a green subpopulation (type B). Above 28 μM, there was no longer an apparent differential response that would otherwise enable discrimination of AI-2 concentration; based on the consistency with modelled behaviour, coculturing contributed to dampen the response as the maximum percentage of responding cells in cocultures is 50% instead of 100%. However, the overall fluorescence output is enriched by the combination of multiple populations since the ranges of sensitivity overlap and effectively expand that of the master population (Supplementary Fig. 8d). Specifically, because the fluorescence of B is described by a larger saturation constant, its fluorescence continually increases at higher AI-2 concentrations, while the fluorescence of A remains unchanged. Thus, coculturing between A and B enables resolvable output that is lower than the detection limit of B (due to A) yet surpasses the upper limit at which A saturates by the inclusion of B. The choice to fluorescently differentiate A and B was important because the output would otherwise be biased by extracellular components including the existence of non-sensing cells. Due to colour designation of A and B, a colour ‘pattern' emerges as a feature of the parallel response, which we recognize is independent of the absolute fluorescence of the population.

### Consensus feedback through multidimensional processing

**Figure 6:**
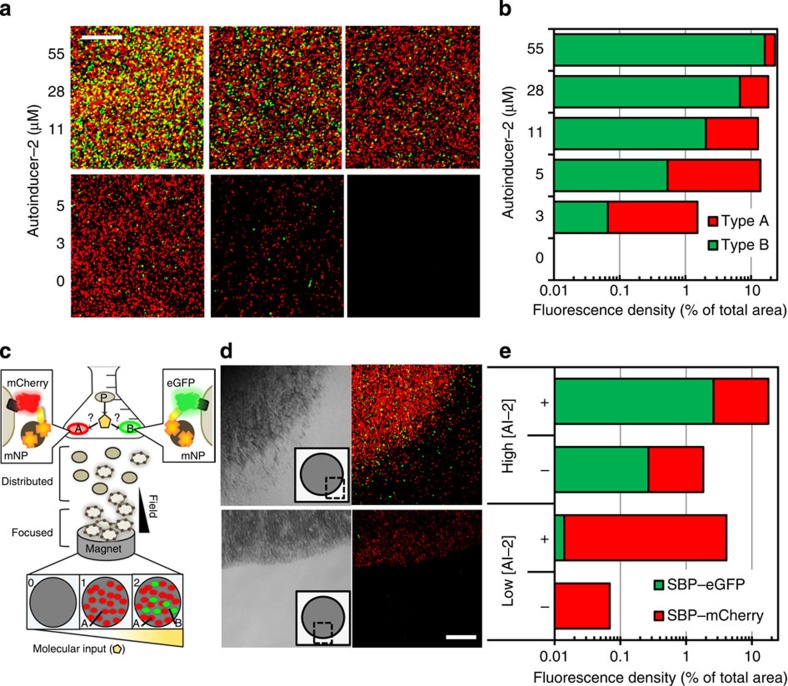
Binning molecular information through cell-based parallel processing and magnetically focusing fluorescence into collective consensus output. (**a**) A and B cell types were co-incubated with AI-2 levels ranging from 0 to 55 μM AI-2 (left axis), then imaged after magnetic nanoparticle coupling and magnetic collation. Fluorescence results (centred directly over the magnet) are shown from high to low input (top left to bottom right). (**b**) Quantification of red and green fluorescence cell densities per AI-2 level. (**c**) The process of accessing molecular information begins by distributing Responders A and B within the environment of an AI-2 producer, P. A and B independently express fluorophore fusions and are linked with magnetic nanoparticles on processing autoinducer-2. Magnetic focusing translocates fluorescing responders. Image analysis of the magnetically collated cell aggregate reveals classified fluorescence output, representing the AI-2 composition of the interrogated environment. (**d**) Bright field (left) and fluorescence (right, red and green filters) images positioned over the edge of a magnet, as indicated by the inset. The sample in the bottom image pair was isolated from an environment of low AI-2 accumulation. The sample in the top image pair was isolated from a high AI-2 environment. (**e**) Quantification of visual space occupied by collated cells (eGFP and mCherry expressers) while distributed (- magnet) and magnetically focused (+). Scale bars, 50 μm.

We hypothesized that the value of cell-based sensing would be enhanced if the cell output could be collated in an unbiased manner that in turn were easily ‘read' using optical means. We engaged magnetic processing, which represents an abiotic processing step that enhances the signal by focusing the collective response. Hence, cells were equipped with streptavidin-conjugated mNPs (Fig. 3). The ability of a magnetic field to refine fluorescence output through filtering and focusing is described in the Supplementary Information (Supplementary Fig. 11). Thus, in our combinatorial approach, fluorescence feedback about molecular information within a microbial community entails biotic processing through constituencies of two independent cell types in conjunction with magnetic post-processing that is enabled by guidance at the nanoscale (Fig. 6c). Moreover, since the fluorescence feedback data is provided through two constituencies, consensus from each independently provides an aggregate output; in our example, the output becomes relayed as a distinctive ‘binned' category due to finite colour-combinations generated from constituencies A and B (Fig. 6c).

Again, type A transmits red output (SBP–mCherry^+^) and type B transmits green (SBP–eGFP^+^). These were first co-incubated with titred concentrations of AI-2, to obtain results similar to those of Fig. 5d. By coupling mNPs to the responsive parallel populations, we tested for aggregate two-colour output to provide informative feedback within a set of outcomes ranging from no colour, red-only to red+green. After overnight co-incubation and a magnetic sweep with streptavidin-mNPs, fluorescence results are shown in Fig. 6a, where the recovered cells are displayed above a magnet's center in order from highest to lowest AI-2 level (top left to bottom right). The processing output generated by the range of conditions was quantitatively assessed for contributions from A and B responders. The spatial density of each fluorophore, or the area occupied by fluorescent responders as a percentage of total visible area, was quantified and plotted in Fig. 6b. Here the trend of increasing fluorescence with AI-2 is followed by both A and B cell types; however, red A cells accumulate at a higher rate than green B cells. This relationship between A and B processing is not only consistent with their previous characterizations (Fig. 5) but indicates that the aggregate output is unbiased regardless of assembly with mNPs and magnetic-stimulated redistribution (Supplementary Information, Supplementary Fig. 14a).

Next, A and B cells were added together to probe the QS environment of *Listeria innocua*, an AI-2-producing cell type that is genetically and ecologically similar to the pathogenic strain *L. monocytogenes*^52^. The environment was biased towards low and high cell density conditions by altering nutrient levels to develop contrasting scenarios of AI-2 level. Preliminary characterization in the Supplementary Information indicated that *L. innocua* proliferation is unperturbed by the presence of *E. coli* responders (Supplementary Fig. 12) and that type A cells detect AI-2 at low *Listeria* densities limited by sparse nutrients; then with rich nutrient availability, cell proliferation permits a higher AI-2 level that can be detected by type B (Supplementary Fig. 13). Replicating these conditions, we expected red fluorescence to be observed at low culture density and for green fluorescence to be reported when high (Fig. 6c). Two conditions were tested: *L. innocua* was proportioned to responder cells at 20:1 in dilute media to establish a low culture density condition or, alternatively, a ratio of 200:1 in rich media for a high culture density condition. After overnight co-incubation and a magnetic sweep (applied directly to the triple strain cultures) with streptavidin-mNPs, the recovered cells are displayed above a magnet's edge (shown in Fig. 6d). Acute focusing of the fluorescence signals, contributed by each subset population of the processor (A and B), is visually apparent. The magnetic field had a physical effect of repositioning the ‘on' subsets to be tightly confined within the magnetic field.

The processing output generated by the contrasting culture conditions was again assessed for the respective contributions of A and B, and for changes in spatial signal density due to the magnetic sweep (Fig. 6e). The analysis was based on images provided in Supplementary Fig. 14b. Data in Fig. 6e indicate that red type A cells are prevalent regardless of culture condition (except negative controls). However, compared with the low AI-2 condition, the abundance of green cells is 100-fold higher in the high AI-2 condition. In addition, the ratio of green to red was consistent prior to and after magnetic concentration, substantiating observations in the distributed system. Further, data show that magnetic refining increased per-area fluorescence 100-fold or 10-fold in low and high cell culture studies, respectively.

Based on the thresholds established for responder populations A and B, we found colour-coded binning corresponded to AI-2 level, where ‘red-only' represented less AI-2 than ‘red+green' (Fig. 5d). Thus, we found a binned output was established via this multidimensional molecular information-processing system and that this matched the expectations. Red feedback (from responder A) indicated dilute AI-2 accumulation occurred in the low density culture. In the dense cultures, high AI-2 accumulation turned on both A and B for combined red and green feedback.

### System response patterns defined by parallel populations

**Figure 7:**
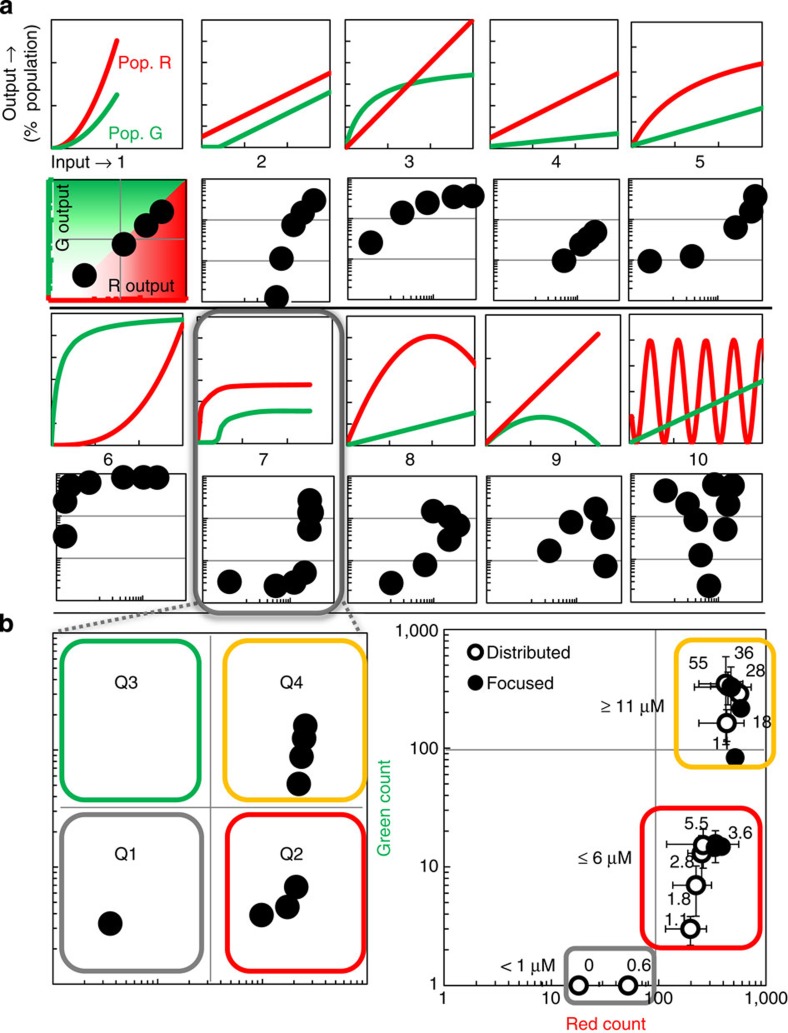
Extension of nano-guided cell networks for hypothetical regulatory structures. (**a**) Rows 1 and 3 depict 10 hypothetical genetic responses to molecular inputs for pairs of fluorescence-reporting cell populations (red, R and green, G). Rows 2 and 4 depict genetic responses as phase-plane plots yielding distinct patterns. This establishes a visual field, showing the extent of any population–population bias (illustrated in example case 1). (**b**) Left panel: a two-population pairing (shown in case 10) defines visual output that inherently bins into three quadrants: Q1, negligible colour; Q2, red bias due to majority red cell output; and Q4, combined red and green output. Right panel: data from Figs 5d and 6b are plotted analogously, where each data point represents an autoinducer-2 input (labelled, μM). As expected, red and green outputs were binned into Q1, Q2 and Q4 as indicated by coloured outlines.

Our example demonstrates the concept of an amorphous processing system that utilizes several biotic and abiotic components for multidimensional information processing. Interestingly, a binning effect was enabled: our system yields an index of colour-categorized feedback that characterizes the sampled environment. In Fig. 7, we present a means to extend our approach to multidimensional systems, those with more than one molecule-of-interest and at different concentrations. That is, by appropriate design of the cell responders, we can further enrich the methodology, its depth and breadth of applicability. We depict 10 hypothetical pairs of responses (with defining equations located in Supplementary Table 5)—those that can be driven by appropriately engineering cells to portend altered genetic responses. For example, rows 1 and 3 provide genetic outcomes as a function of analyte (AI-2) concentration. The hypothetical depictions are feasible as ‘designer' signal transduction and marker expression processes enabled by synthetic biology^21^,^53^,^54^. Rows 2 and 4 demonstrate the corresponding visual planes, where red cell numbers (*x*-axis) are plotted against green (*y*-axis), illustrated by the first example. If one divides the two-dimensional space into quadrants (no colour, majority red, majority green, and equivalent ratios of red and green), it becomes apparent that the relationship between cell types influences the ‘visual' or optical output. Thus, the 10 arbitrary response sets yield a variety of pairings that can provide unique visual patterns for categorizing molecular information. We have simplified the analysis by placing dot marker symbols at the various coincident datapoints, revealing visual patterns. In this way, the ability to incorporate unique responses to a multitude of molecular cues, all within a single pair of cells, or through further multiplexing with additional cell populations becomes apparent.

Our AI-2-conveying cell network is similar to example 7 in Fig. 7a and the AI-2 response curves in Fig. 5 (characterized by Supplementary Table 4 equations). Example 7 establishes output into three basic quadrants, including Q1 (negligible colour), Q2 (majority red) and Q4 (roughly equal red and green) (Fig. 7b). We recast the data from Figs 5d and 6b as a phase-plane portrait in Fig. 7c. This reveals the mechanisms by which the output is binned and how the originating cell response curves lead to this pattern, which in turn, was unchanged due to magnetic refinement. In Supplementary Fig. 15, we demonstrate a parameterization of the red and green response curves that suggest the methodology is robust, that when cells are appropriately engineered one could ‘tune' system characteristics to enhance or diminish a binning effect. We suggest that the utility of subcellular genetic tuning extends well beyond per-cell performance. Rather, we suggest such a strategy may be used to guide the dynamics of population architecture for actuation of by-design response patterns at a systems level.

## Discussion

While cell-based sensors work well in well-defined assay conditions, extension to complex environments remains a challenge. They grow, they move, they perturb their environs, they report in a time and concentration-dependent manner, small numbers of sensor cells may require signal amplification and so on. Also, increasingly, bacterial cells are engineered for user specified ‘executive' functions in complex environments^55^,^56^,^57^. Their performance depends on their ability to filter out extraneous noise while surveying the molecular landscape, and providing informed actuation.

Our system interrogates the molecular space by focusing on bacterial QS and a widely distributed signal molecule, AI-2. In addition to genetic attributes of the AI-2-responding sensor cells, AI-2 is a chemoattractant for *E. coli*, and hence *E. coli* engineered to sense and respond to AI-2 will naturally move towards its sources, enabling full sampling of the prevailing state^10^,^37^. Each strain evaluates AI-2 with a distinct sensitivity. When ‘activated' in response to a characteristic level, the cells simultaneously expressed a fluorescent marker and a SBP on the outer membrane via AIDAc translocation. SBP provides a means for cell hybridization through its strong affinity to streptavidin, and here, aids in binding mNPs. This enables the non-genetically coded property of cell translocation within a magnetic field through physically stimulated focusing and binning.

By making use of a diversity of biotic and abiotic features, our multidimensional system of ‘responder' populations exemplifies several key metrics that promote executive performance in such environments: active molecule capture, post-capture refining of the detection output and finally the utilization of multiple feedback thresholds^58^,^59^,^60^. Here cells facilitate AI-2 recognition autonomously and actively because, as a distributed network they reside planktonically, chemotaxing to and continually processing signals over time. When AI-2 is detected, a processor cell's cognate machinery responds by upregulation of the native QS operon, leading to rapid signal uptake and thereby creating an active-capture signal-processing mechanism. To maximize information acquisition and account for a potentially heterogeneous molecular landscape, cells serve as molecular sampling units among a distributed population, which leads to data fed back as a consensus of fluorescent ‘datapoints'. Then, distributed data collection can be selectively reversed via the incorporated abiotic feature: mNPs, fastened externally on the cell through affinity-guided self-assembly. As such, responding cells obtained this extendable feature, thereby becomes sensitized to repositioning within a magnetic field.

The layered nature of the processor here, from the subcellular to multicellular scale, permits a series of selective steps: it commences with the AI-2-triggered expression cascade which releases a tight repressor, surface localization of both the fluorescent protein and SBP tag, and finally nanoparticle binding for recovery. In addition, multiple layers of amplification result in orthogonal fluorescence feedback. The AI-2 detection event leads to whole-cell fluorescence through expression of many protein copies^47^. Then their physical collection further amplifies the signal, yielding a macroscopic composite of many individual cell units. When utilized as a network of multiple constituencies, responder cell types A and B contribute individual recognition results (off, red or green) to a single consensus output. Finally, due to their overlapping thresholds for recognition of the same molecule, in this case, AI-2, parallel processing by A and B responders can contribute to visual interpretation of information about the molecule. Outcomes are classified into a finite number of states: here output to no fluorescence, red, or red and green, with each addition of colour as a metric of a higher interval of AI-2. In many respects, the elucidation of layered information networks as demonstrated here is analogous to computer information processing via information theory^61^,^62^,^63^.

Here, however, interrogation of biological systems requires a reliable means for accessing molecular information—that which is communicated between biological species and that which can be relayed to the end user. The responder cells need not be present in high concentration, nor must they all be collected in the present format. We suggest that engineered biological mechanisms are well-poised to serve at this critical interface between information acquisition and user interaction. Thus, the functional design of components for autonomous self-assembly, decision-making and networking is requisite in the field of micro- and nano-scaled machines. Our combinatorial approach allows for cells to independently assess, yet collectively report, on molecular information. Its processing is enabled through appropriate integration of synthetic biology and nanomaterials design. We suggest this approach provides a rich opportunity to direct many formats of multi-population response through genetic tuning and systems-level engineering. Further development of cellular networks and incorporation of alternate abiotic attributes can expand the depth and breadth of molecular communication for user specified actuation.

## Methods

### Engineered strains

All plasmids and strains used in this study are listed in Supplementary Table 1. The vectors designed for this study, pSBP–Venus, pSBP–mCherry and pSBP–eGFP, were derived from pAIDA-I, which was generously donated by Larsson^40^ and previously used for covalent surface display of fusions up to 110 kDa in size. The plasmids pSBP–Venus, pSBP–mCherry and pSBP–eGFP were constructed as described in the Supplementary Information using primers listed in Supplementary Table 2 and the gene sequences of Supplementary Table 3 as templates. The resulting plasmid constructs are mapped in Supplementary Fig. 1. Plasmids were transformed into chemically competent BL21(DE3) *E. coli* (Life Technologies) for testing T7-regulated expression of the surface display fusion SBP-fluorescent protein-AIDAc. Next, the plasmids were introduced by electroporation into the electrically competent strains CT104 (+pCT6) and MDAI2 (+pCT6). Strains were made competent by standard procedures.

### Protein expression and labelling

Chemically competent BL21(DE3) cells (Life Technologies) were transformed with pSBP–Venus, pSBP–eGFP or pSBP–mCherry. Cultures were grown to mid-exponential phase, then induced with 500 μM isopropyl B-D-1-IPTG, purchased from Sigma (USA). The induced cells were incubated at 37 °C, 250 r.p.m. shaking, for 6 h. Alexafluor488- and Alexafluor594-labelled streptavidin (Life Technologies, #S-11223 and S-11227) were prepared to a working concentration of 20 μg ml^−1^ in 10 mM PBS. Dylight405-labelled streptavidin was prepared from a ThermoScientific labelling kit and diluted to a working concentration of 500 μg ml^−1^. Culture aliquots were washed once in PBS, centrifuged (4,000*g*, 5 min), resuspended in the fluorescent streptavidin solution and labelled for 1 h at room temperature. Finally, cells were again washed in PBS and resuspended for imaging. Imaging parameters for each fluorophore were consistent for each composite. To instead immunolabel surface-expressed eGFP, induced cells were first washed in 10 mM PBS and incubated with anti-GFP monoclonal mouse IgG (Rockland Immunochemicals, #600-301-215), diluted 1:100 in PBS for 1 h at room temperature. Samples were washed twice in PBS, then incubated with Alexafluor594 (red) anti-mouse polyclonal goat IgG (Molecular Probes, #A-11032) as a secondary antibody, diluted 1:200 in PBS, again for 1 h at room temperature.

### Cell culture

*E. coli* cultures were prepared by 1% reinoculation into LB medium from an overnight culture and grown at 37 °C, 250 r.p.m. shaking, until mid-exponential phase, at which point experiments for triggered expression were initiated. Antibiotics were added according to the plasmids contained by the strain. Ampicillin was used at 100 μg ml^−1^ for pCT6 transformants and kanamycin at 50 μg ml^−1^ for pSBP transformants. CM were isolated from W3110 *E. coli* by pelleting culture aliquots and by filter-sterilizing the supernatant. *L. innocua* cultures were inoculated into a brain–heart infusion (BHI) medium and grown at 30 °C, sampling at cell densities specified in results. For cocultures with *L. innocua*, all cell types were centrifuged at 4,000*g* for 5 min and resuspended at the same density into antibiotic-free medium, varied according to the experiment. To initiate cocultures, the reporter cells CT104 (Type ‘A') and MDAI2 (Type ‘B') were added to *L. innocua* at 1% each to a 48-well plate. Cocultures were then grown at 30 °C, 220 r.p.m. shaking, overnight for 12–14 h. *L. innocua* was prepared for coculture by isolating the strain at OD intervals during growth and resuspension. For coculturing with CT104*, L. innocua* was grown to OD 0.3 and then resuspended in diluted media: 2.5% BHI (7.5% LB, 90% PBS); in addition to OD 0.3, cells were diluted 10- and 100-fold before coculturing. For coculturing with MDAI2, *L. innocua* was grown to OD intervals between 0.05 and 0.6 and the media was replaced with 25% BHI in LB. Initial cell number ratios were obtained by colony count calculations from plated cells.

### Western blot

Samples were prepared by incubating cell cultures at 30 °C, 250 r.p.m. shaking in LB broth with 100 μg ml^−1^ ampicillin and 50 μg ml^−1^ kanamycin. Samples were induced with either 28 μM AI-2 and incubated overnight (for comparison across fluorescence protein variants) or 20 μM AI-2 for timecourse analysis. At the designated timepoints, samples were pelleted and washed twice in cold PBS. For cell lysis, the pellets were resuspended in Bugbuster (EMD Millipore) at 5% of the original volume (per 3 × 10^9^ cells per ml) at room temperature for 1 h. The lysis mixture was then centrifuged to separate the cell membrane fraction (in pellet) and cytoplasmic fraction (supernatant). The pellet was washed twice in PBS, then resuspended in 10% SDS at an equal volume to the supernatant fraction. Samples corresponding to 6 × 10^8^ cells (either the supernatant or pellet fraction) were loaded into 10% acrylamide/bis-acrylamide (37.5:1, BioRad) gels with a protein ladder (Benchmark, Life Technologies) and run at 200 V for 45 min. The gels were transferred to nitrocellulose membranes (BioRad) at 10 V followed by 20 V for 20 min each. The gel was then stained with Coomassie blue and destained to visualize retained protein and the nitrocellulose was blocked overnight at 4 °C in 5% milk-TBS. The membrane was probed for SBP-tagged proteins using alkaline phosphatase-conjugated streptavidin (Life Technologies, #S-921) at a 1:1,500 dilution in 1% milk-TTBS. Finally, protein bands were determined using NBT/BCIP (Roche) as a colorimetric substrate for alkaline phosphatase. Uncropped blots are presented in Supplementary Fig. 5.

### Flow cytometry

Fluorescent cell counts for SBP–Venus reporters were obtained using a FACSCanto II flow cytometer (Becton Dickinson), the 488 nm laser and 530/30 green filter and BioFACS Diva software. Histogram images (Supplementary Fig. 4c) were obtained using FlowJo software (Tree Star). Aliquots from culture were diluted 4-fold in PBS and counts of at least 30,000 were recorded for each sample. Specifically, 50,000 events were counted in the analysis for Fig. 5b and gated for Venus fluorescence at an intensity of 2 × 10^3^. Supplementary Fig. 13 results were based on subpopulations gated above an intensity of 1 × 10^3^ out of 50,000 events.

### Confocal imaging and analysis

Fluorescence imaging utilized a Carl Zeiss (Jena, Germany) LSM700 confocal microscope. Microscopy images were processed and analysed using ImageJ (the National Institutes of Health). Profiles of fluorescence mean grey values were generated for image slices with approximately one cell per vertical space. Alternatively, the brightness threshold was adjusted uniformly across images, after which a particle analysis was used to count cells. To distinguish cell type within coculture images, a combined analysis counted particles based on size threshold and ratio of green to red intensity. This analysis is described in the Supplementary Information and depicted by Supplementary Figs 9 and 10.

### Scanning electron microscopy

Induced BL21(DE3)+pSBP–Venus cells and streptavidin-conjugated mNPs were prepared separately, as well as mixed together to isolate cells with surface-bound mNPs. MNP-decorated cells were isolated by co-incubating for 20 min on ice, then magnetically recovering a pellet, which contained observable cells. Then samples were fixed and dehydrated. After each step, cells were pelleted by centrifugation at 3,000*g*, 4 °C, 5 min. mNPs and cell-mNP mixtures were pelleted magnetically. Samples were first washed twice with cold PBS, then incubated at room temperature with 4% paraformaldehyde (Sigma-Aldrich) in PBS. Subsequently, the samples were washed once with PBS and twice with deionized water. Next, gradual replacement with ethanol was performed by sequential 10-min incubations on ice with 30, 50, 70 and 100% ethanol in water. Finally, ethanol was gradually replaced with hexamethyldisilazane (HMDS, Sigma-Aldrich) by 10-min incubation in 50% HMDS solution in ethanol followed by two incubations with 100% HMDS. Samples were ventilated on a coverslip in a chemical fume hood for 30 min, and then further dried at 37 °C for 1 h. Prior to imaging, the coverslips were sputter-coated with carbon using a MED 010 evaporator (Balzers Union, Liechtenstein). Sample imaging and elemental mapping used a SU-70 UHR Schottky field emission SEM equipped with an energy-dispersive x-ray spectrometer (Hitachi, Tokyo, Japan).

### Magnetic-activated cell focusing

mNPs (100 nm, fluidMAG-Streptavidin; Chemicell, Berlin, Germany) were washed in cold PBS and resupsended at the same density. For magnetic enrichment of multi-species surveillance systems, mNPs were applied directly to overnight cultures at 2% of the culture volume. After incubating the cells and mNPs in a 96-well plate at 4 °C for 20 min, a 2.2 × 1.6 cm (dia. × ht.) neodymium N42 magnet (K and J Magnetics) was placed under each well for 5 min to collect mNPs on the bottom surface. The collected pellet consisted of magnetically coupled contents; the supernatant was removed and the sample was resuspended in a minimal volume (∼2 μl). To image the particle-coupled contents within a magnetic field, a 1.6 × 0.8 mm (dia. × ht.) N52 neodymium magnet was taped behind a coverslip. About 2 μl of suspended mNPs and cells bound with mNPs was added to the opposite side of the coverslip, directly on top of the magnet, set for 2 min, after which another coverslip sealed the sample for imaging at × 200 magnification. Characterization of streptavidin-coated mNPs for SBP-surface-expressing cells is provided in the Supplementary Information.

## Additional information

**How to cite this article**: Terrell, J. L. *et al*. Nano-guided cell networks as conveyors of molecular communication. *Nat. Commun.* 6:8500 doi: 10.1038/ncomms9500 (2015).

## Supplementary Material

Supplementary Information Supplementary Figures 1-15, Supplementary Tables 1-5, Supplementary Methods and Supplementary References

## Figures and Tables

**Figure 1:**
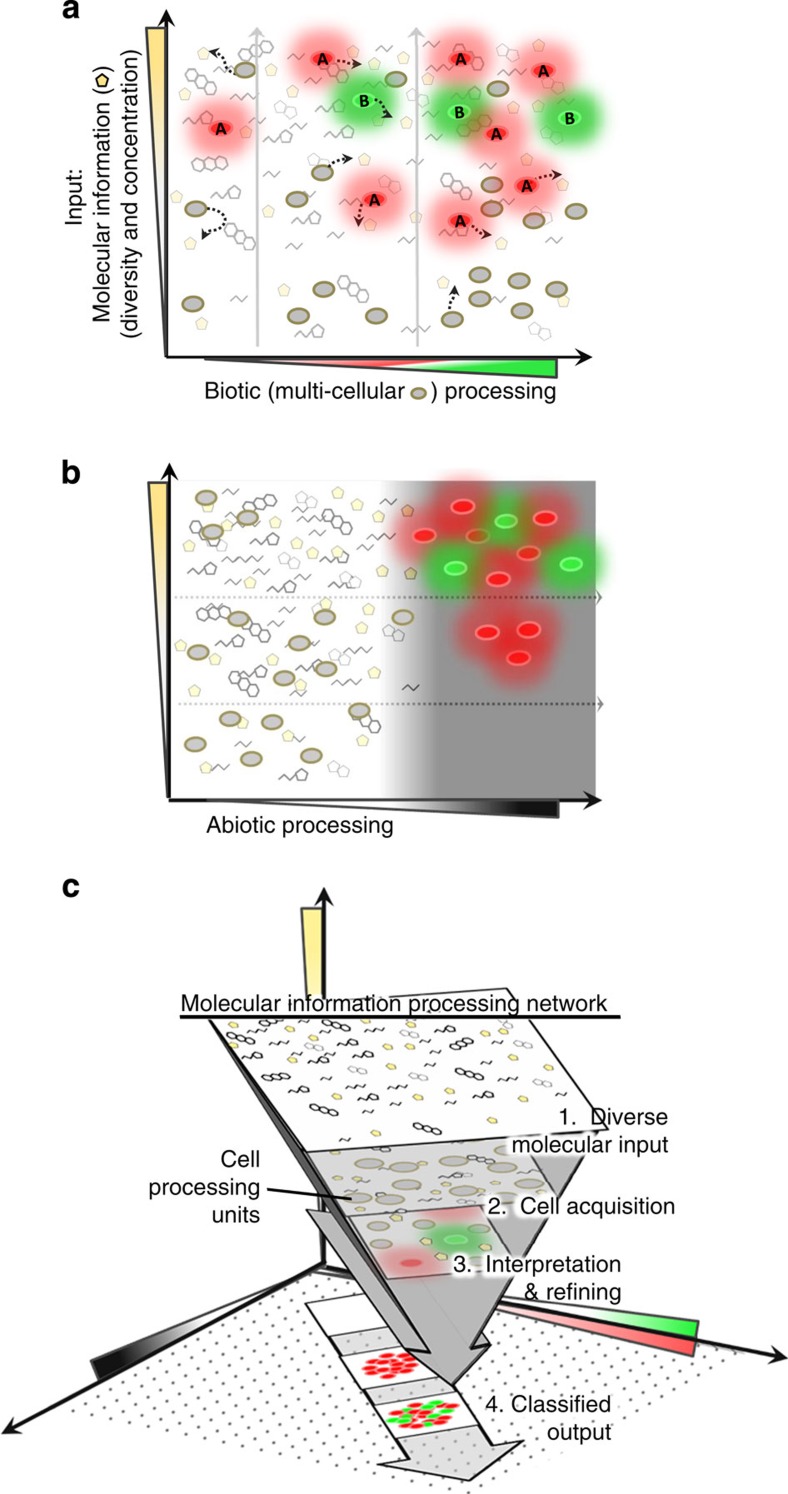
Nano-guided cell networks for processing molecular information. (**a**) Biotic (multicellular) processing is facilitated by cell recognition, signal transduction and genetic response. The genetically encoded response reflects the identity and prevalence of the target molecule(s). Biotic processing includes both increased cell number of responders and their genetically tuned response patterns. (**b**) Abiotic processing, used in conjunction with biotic processing, adds dimensionality to cell-based output by modifying through a physical stimulus (in our example, magnetic focusing). (**c**) Schematic of a cell population and nanomaterial-based network comprising both biotic (green/red axis) and abiotic (black axis) processing mechanisms. This conceptual system interprets molecular information by intercepting diverse molecular inputs, processes them autonomously through independent cell units within the system and refines output to include positive responders that are viewed via orthogonal means (visual classification). The system's hierarchical structure allows molecular information to be refined into categorized collective outputs.
