# Supplementary material for: Nano-guided cell networks as conveyors of molecular communication
Source: Nat Commun. 2015 Oct 12;6:8500. doi: 10.1038/ncomms9500 (PMC4633717; doi:10.1038/ncomms9500)
Supplement: Supplementary Information — Supplementary Figures 1-15, Supplementary Tables 1-5, Supplementary Methods and Supplementary References [file ncomms9500-s1.pdf]

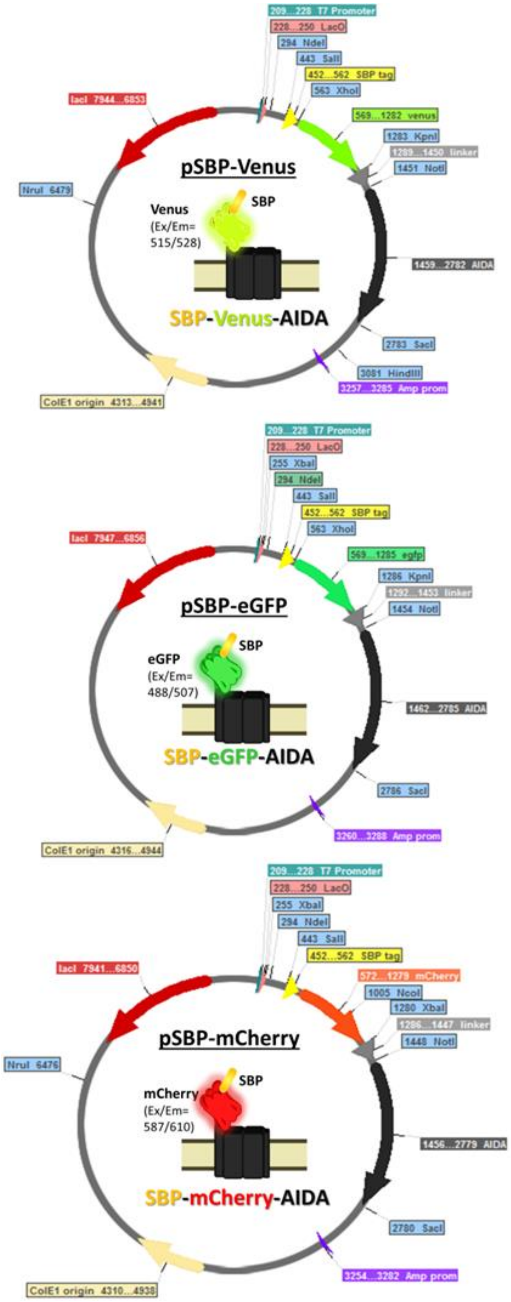

Supplementary Figure 1 AIDAc-tethered surface displays fused with fluorescent proteins and a streptavidin binding peptide (SBP) tag and T7-plasmid constructs. Representations are depicted above of the outer membrane containing the AIDAc transmembrane domain with either Venus-SBP, eGFP-SBP, or mCherry-SBP expressed on the outer surface.

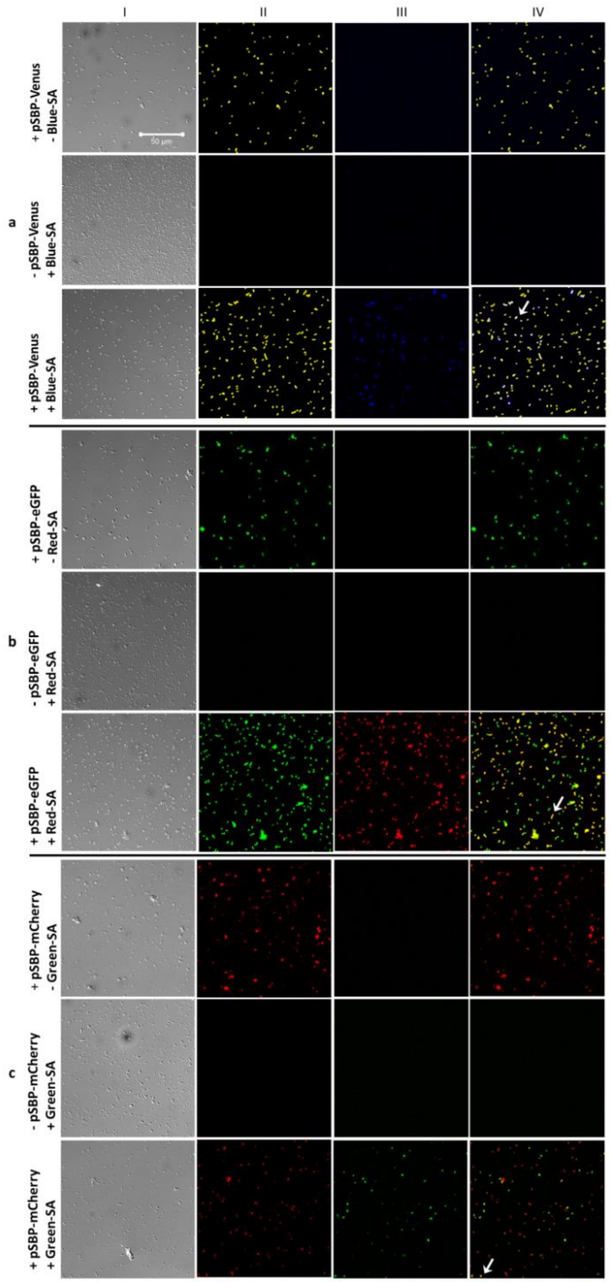

Supplementary Figure 2 Streptavidin-labeling of SBP to assess its surface accessibility. Fluorophore-tagged streptavidin (SA) was used to probe for SBP expressed on the outer membrane of BL21(DE3). Each of three composites represent a set of controls, BL21(DE3) expressing the SBP-fluorescent protein fusion but unlabeled with streptavidin (row 1) and labeled with streptavidin but without the fusion gene (row 2); additionally, both the conditions of fusion gene expression and streptavidin labeling are positive in row 3. Columns I-IV represent each filter applied to the image: I, brightfield; II, filter for the fluorescent protein; III, filter for the fluorescent streptavidin; and IV, an overlay of II and III. Fluorophores with non-overlapping spectra were paired. **(a)** Venus expression (yellow-green) was paired with Dylight405-labeled SA (blue). **(b)** EGFP expression (green) was paired with Alexafluor594-labeled SA (red). **(c)** mCherry expression (red) was paired with Alexafluor488-labeled SA (green).

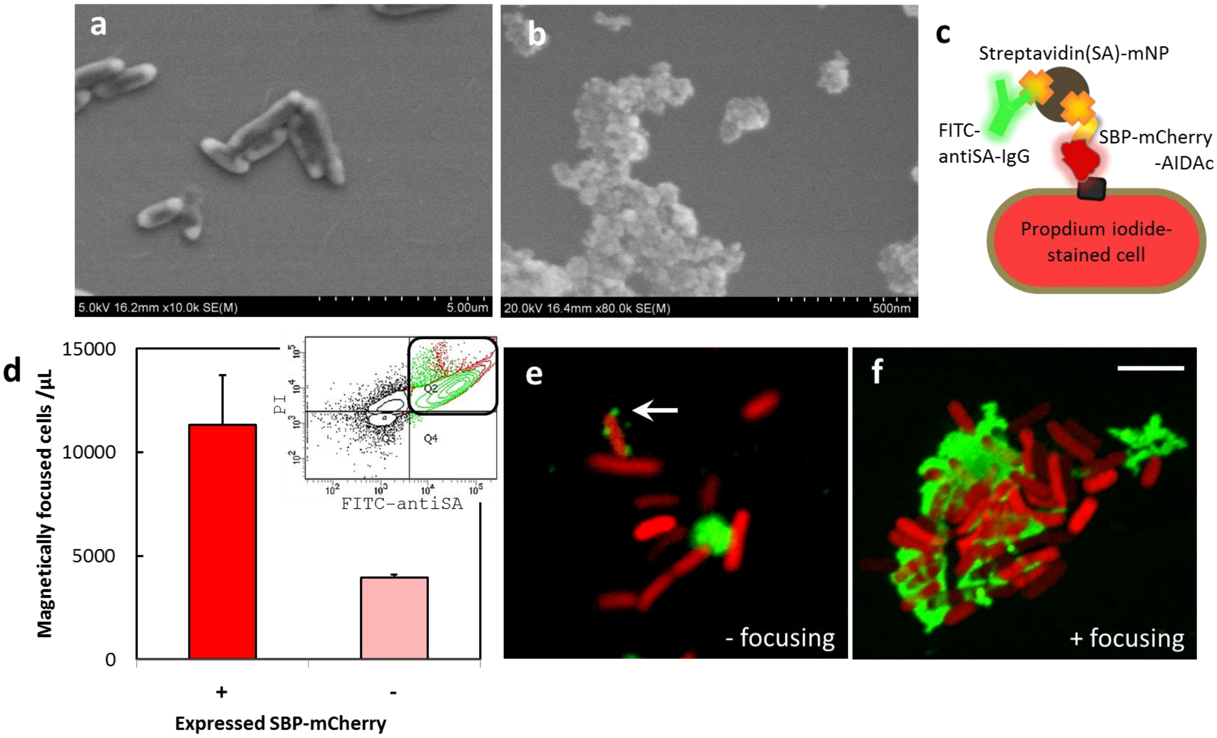

Supplementary Figure 3 Analysis of cell interaction with streptavidin-coated magnetic nanoparticles. Electron micrograph of (a) *E. coli* K12 cells and (b) streptavidin coated magnetic nanoparticles (mNPs). (c) scheme of immunoprobed cell-mNP complexes. (d) Comparison between recovery via mNP-coupling and magnetic collection for cells expressing surface-displayed SBP-mCherry (+) or non-expressing cells (-). Data is in triplicate and statistically significant across controls with $p < 0.05$, deterimined by a Student's t-test. Inset FACS distribution of fluorescence intensities maps the red (propidium iodide-stained) cells that are bound with FITC-anti-streptavidin (SA) labeled mNPs (circled in Q2). (e) X magnification of red cells, some of which have assembled with mNPs (example indicated by arrow). (f) Composition and organization of cell-mNP complexes after the application of an external magnet used to focus the hybrid assemblies. Scale bar indicates 5 µm.

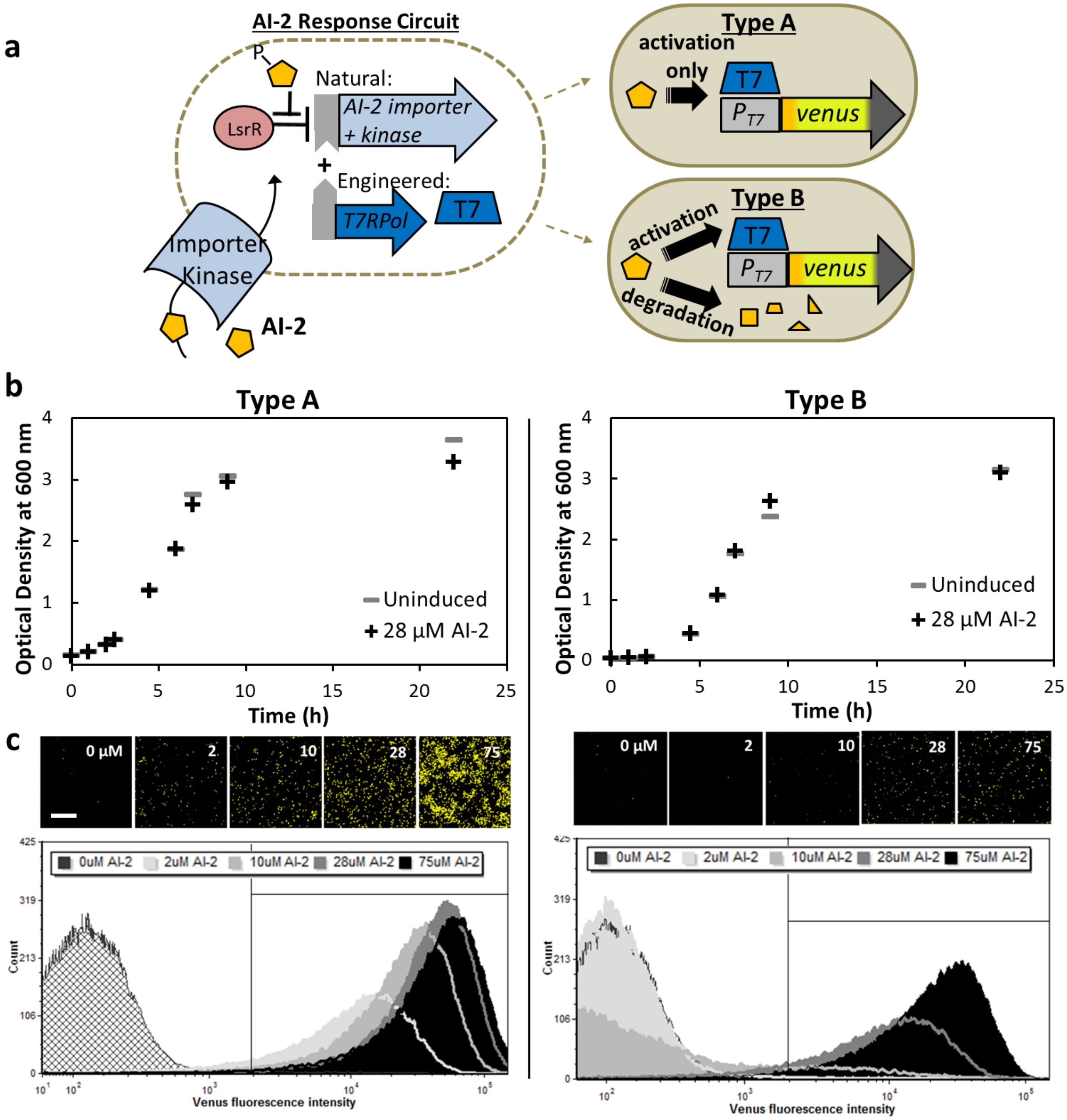

Supplementary Figure 4 Engineered "Responder" cells for autonomous response to the small molecule autoinducer-2. (a) Two host strains CT104 (Δ*luxS*, Δ*lsrFG*) and MDAI2 (Δ*luxS*) were engineered for Type A and B, respectively, by linking marker chimeric protein expression to the natural response pathway for AI-2. (b) Growth curves of responders Type A and B (+ pSBP-Venus) upon addition of 28 µM AI-2 (at induction point represented by dotted line) compared against an uninduced culture. Optical density at timepoints was measured at 600 nm. (c) Fluorescence images of Responder Type A and B culture samples incubated with a range of *in vitro*-synthesized autoinducer-2 (AI-2) concentrations and corresponding FACS gating for population analysis of SBP-Venus expression. Scale bar indicates 50 µm.

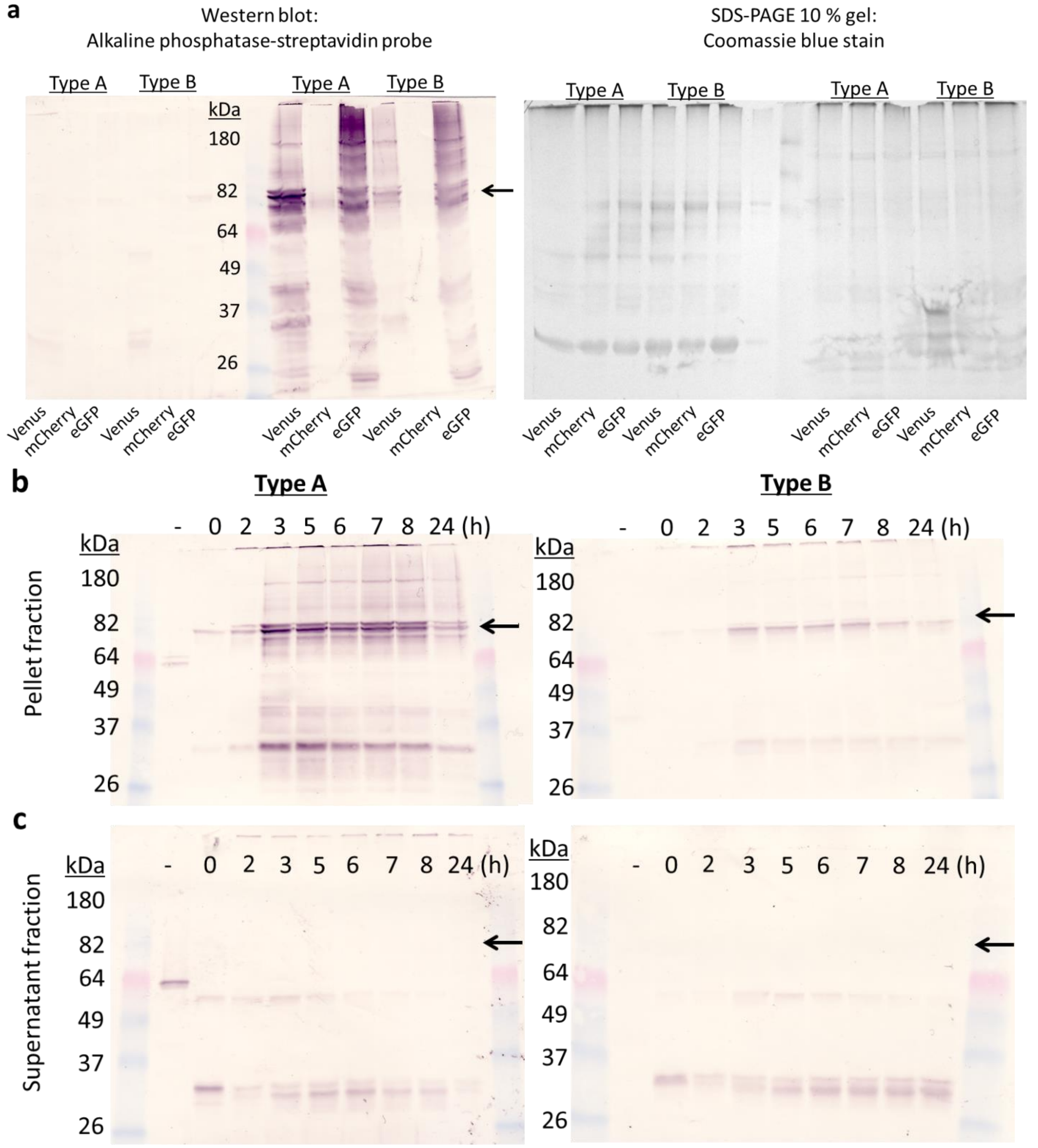

Supplementary Figure 5 Western blot analysis of SBP-fluorophore-AIDAc membrane expression. Proteins isolated from Type A and B cells were size-separated by SDS-PAGE and blotted, using alkaline-phosphatase-linked streptavidin to probe for SBP-tagged proteins. (a) Western blot and corresponding SDS-PAGE gel for cytosolic (left of ladder) and membrane fractions (right of ladder) of induced cell extracts. Western blots for (b) pellet fractions and of (c) supernatant fractions for Type A and B samples isolated at time intervals after induction with 28 μM AI-2. Arrows indicate the position of the 82 kDa SBP-fluorophore-AIDAc fusions.

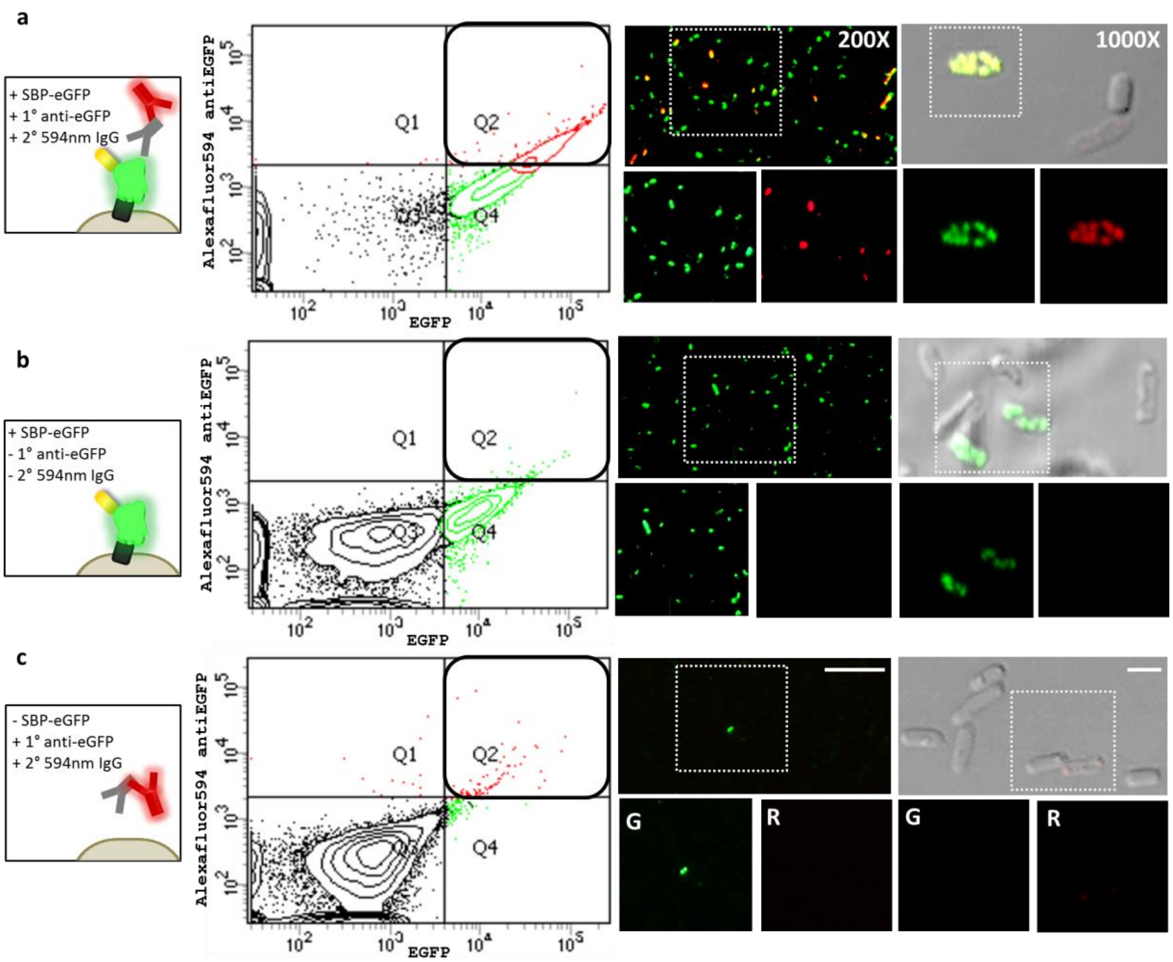

Supplementary Figure 6 Immunolabeling the fluorescent protein to assess surface accessibility. The external surfaces of cells expressing AIDAc-eGFP-SBP were probed with an anti-eGFP and Alexafluor594-labeled antibody pair. Colocalization of green (eGFP) and red (surface immunostaining) was assessed by flow cytometry and microscopy. Each row shows included controls: (a) cells expressing the eGFP fusion and antibody-treated, (b) eGFP-expressing cells without antibody treatment, and (c) non-expressing cells treated with the antibody. Data across each row includes: A scheme of the experimental set-up. FACS distribution of fluorescence intensities, approximated into quadrants – Q1 red cells, Q2, cells exhibiting red and green, Q3, non-fluorescent cells, and Q4 green cells. 200X magnified fluorescence image showing overlaid red and green filters, with the boxed area split into the green (G) and red (R) filter. 1000X magnified transmitted light image with overlaid red and green fluorescence, with boxed area split into the green (G) and red (R) filters. Scale bars indicate 25 μm and 2 μm, respectively.

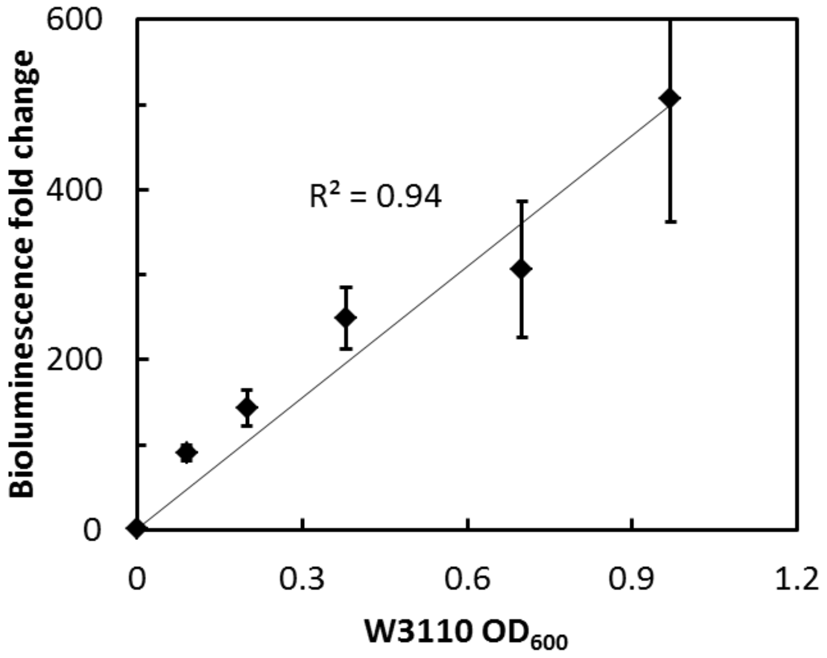

Supplementary Figure 7 AI-2 activity reported by standard *Vibrio harveyi* bioassay. Bioluminescence units were measured from *V. harveyi* strain BB170 response to AI-2 and fold changes were normalized to a negative control. Samples consisted of conditioned media isolated from a W3110 culture during growth, correlated to the OD_{600} measurement at the time of sampling. Standard error of replicates and linear correlation of data is shown.

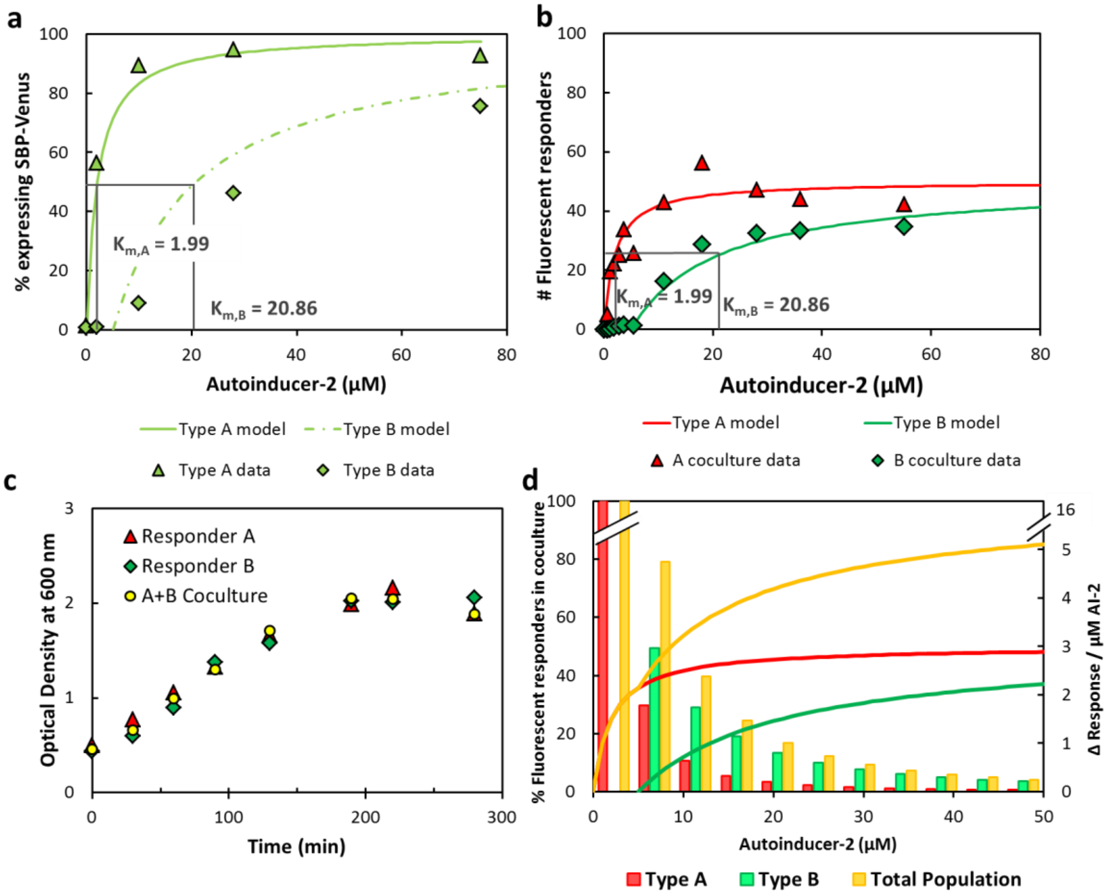

Supplementary Figure 8 Analysis of AI-2 response by Type A and B populations in coculture. (a) FACS data overlaid with Michaelis-Menten or Monod-type models describing population percentage expressing SBP-Venus as a function of AI-2 concentration. (b) Population models with coculture-adjusted parameters (describing responding A and B number per 100 total cells) overlaid with relative cell numbers of red A and green B in coculture for an AI-2 concentration range. (c) Optical density, measured at 600 nm, of Type A and B cultures, isolated and cocultured (1:1 volumes A to B), as a function of time post-induction with 25 µM AI-2. (d) Modeled coculture response from A and B, described by (b), plotted along with their sum representing the total response from the master population. Additionally, the resolution of each curve (change in response per µM AI-2) is plotted for intervals between 0 and 50 µM AI-2.

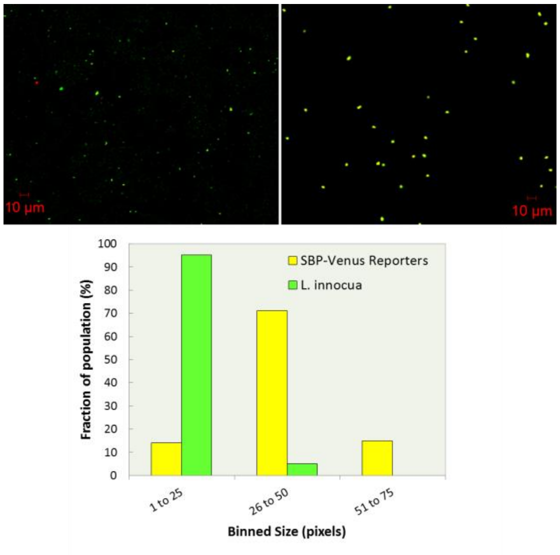

Supplementary Figure 9 Image analysis of particle size from both Syto-9 stained *L. innocua* and SBP-Venus reporter fluorescence.

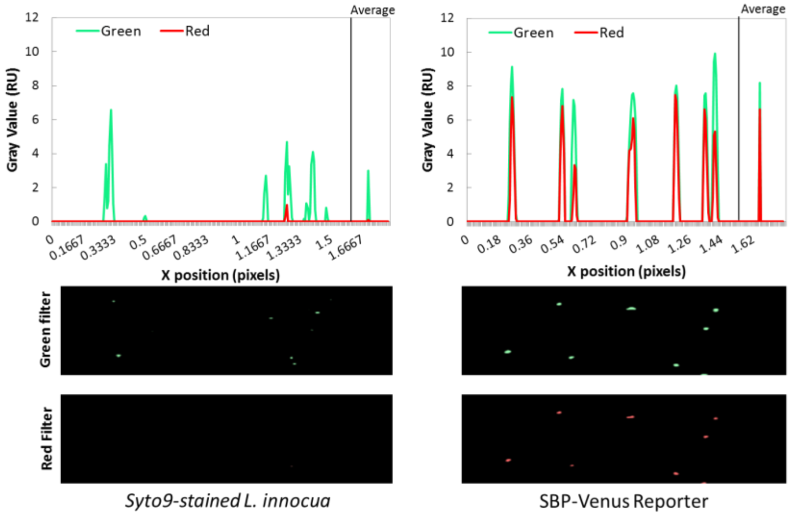

Supplementary Figure 10 Image analysis of red and green pixels from both Syto-9 stained *L. innocua* and SBP-Venus reporter fluorescence.

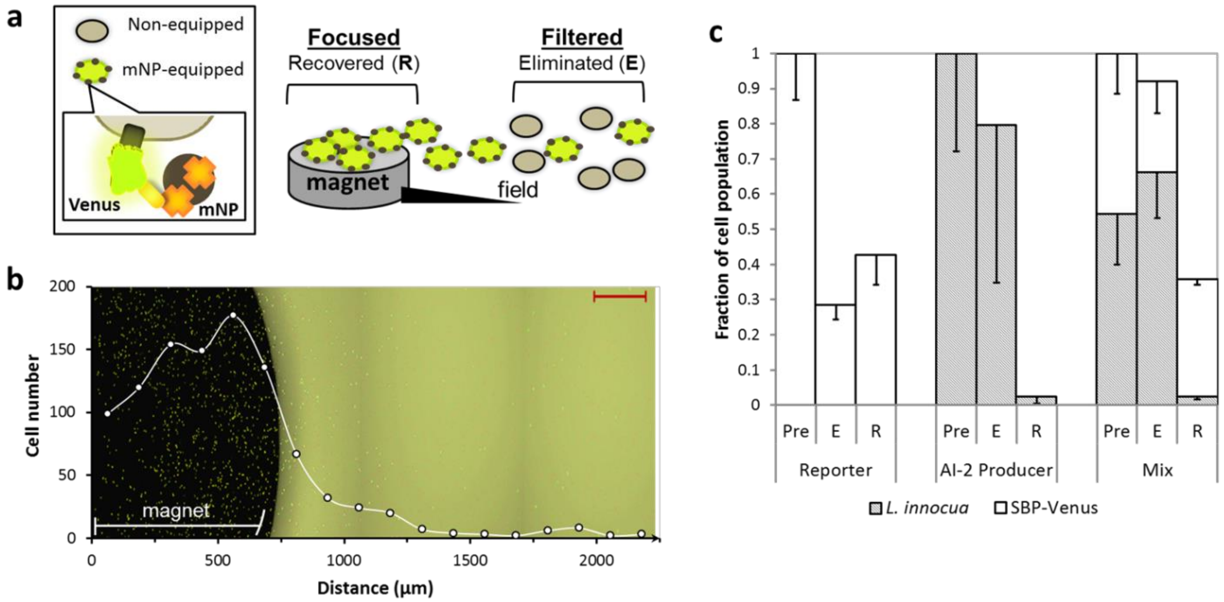

Supplementary Figure 11 Filtering magnetically-equipped cells from AI-2 producers in order to focus fluorescence within a magnetic field. (a) Magnetic refining was performed applying a magnet to collect SBP-expressing cells equipped with streptavidin-functionalized mNPs into a pellet. Both the focusing and filtering properties of a magnetic field were investigated. (b) Observations of magnetic focusing were visualized by applying a mixture of cells and mNPs directly on top of a coverslip containing a magnet beneath. Tiling was used to compile an image of SBP-Venus expressers over 1 mm from the edge of the magnet. Cells were counted and plotted as a function of distance. Scalebar indicates 250 µm. (c) Magnetic filtering was measured by comparing the recovered counts of magnetically equipped cells against the eliminated count for non-equipped cells. Cells were counted from fluorescence images of the separated supernatant (eliminated fraction (E)) and the pellet (recovered fraction (R)), resuspended in an equal volume. The counts were compared in triplicate to the original number of cells in the mixture and reported as a fraction thereof. MACS was performed on SBP-Venus expressers, Syto-9 stained *L. innocua*, and a mixture of the two.

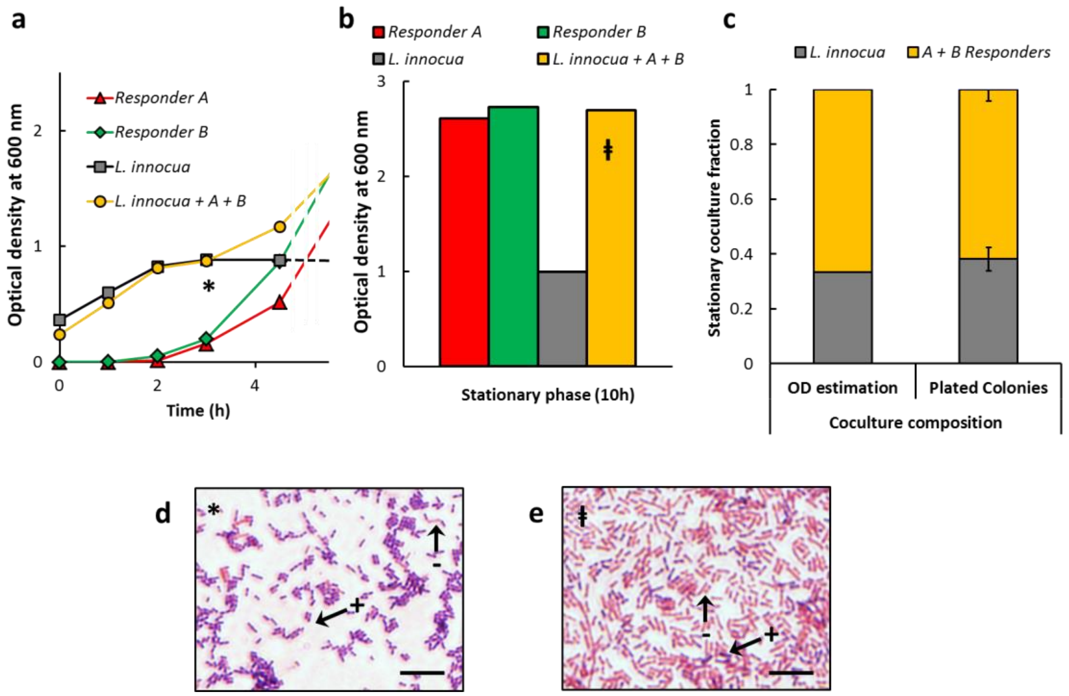

Supplementary Figure 12 Culture growth dynamics in isolated and mixed-species cultures. The growth of *Listeria innocua* in a mixed medium (3:1 Luria broth to brain-heart infusion) was compared against *E. coli* responders A and B when grown separately and when inoculated identically into a mixed-species culture. (a) Optical density measurements (at 600 nm) of cultures during the first 5 h of growth. (b) Final OD measurements 10 h after all cultures reached stationary phase. (c) The final coculture composition was calculated by counting colonies from species-selective plating, plotted from triplicate counts with standard deviations, and compared against the stationary OD of *L. innocua* as a fraction of that of the coculture. (d) and (e) show Gram staining images of the coculture at representative timepoints: 3 h (*) and 10 h (ƚ). Examples of Gram-postive (+) cells, stained violet, and Gram-negative (-) cells, stained pink, are indicated by arrows. Scale bars indicate 5 μm.

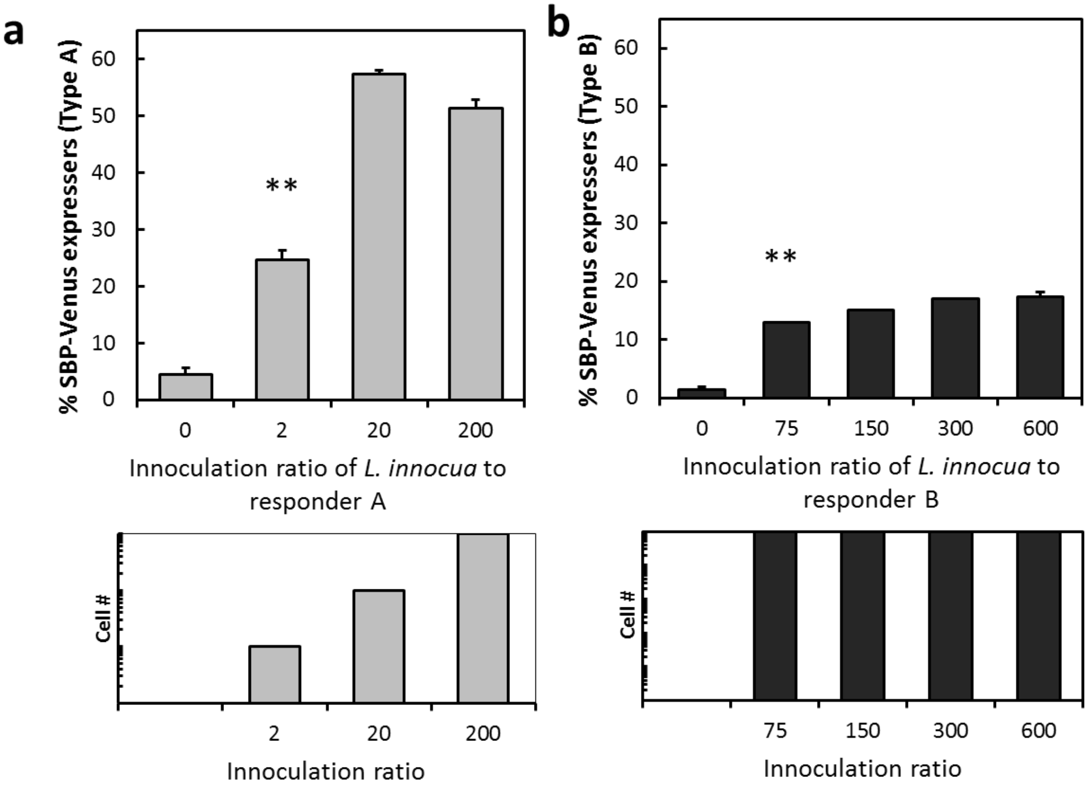

Supplementary Figure 13 Effect of initial culture density on quorum sensing-surveillance of *Listeria innocua*. Upper graphs show Venus expression by populations distributed within cultures of the AI-2 producer *L. innoua*, where the inoculation ratio between the producer and responder cells was varied. Below, final coculture densities are reported below as an order of magnitude. (a) Population A assessed AI-2 prevalence in conditions of limited nutrients and (b) B in conditions of rich nutrients. Symbols denote the onset of statistical significant of triplicate data compared to OD_{600} 0. Statistical significance of data, determined by Student's t-test, is represented by * for $p < 0.05$ and ** for $p < 0.005$.

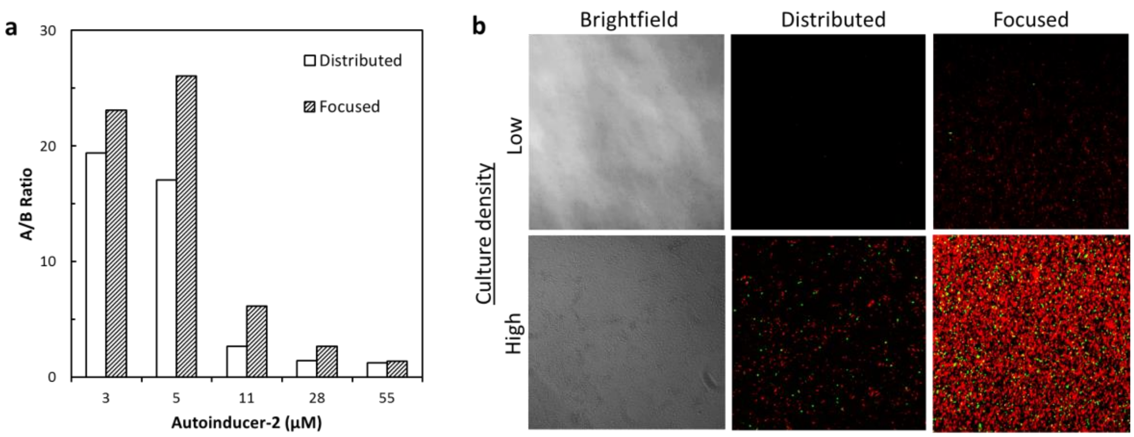

Supplementary Figure 14 Magnetic refining of two-color responding A and B cells from a master population. (a) Ratio of Type A cells expressing SBP-mCherry to B cells expressing SBP-eGFP while distributed in culture and upon focusing to a magnetic surface. (b) Images of visual space occupied as a result of AI-2 information processing in a *L. innocua cu*lture in parallel by AI-2 responder types A (SBP-mCherry +) and B (SBP-eGFP +). Culture conditions were biased between a low and high cell density. Images depict the multi-dimenstional system of *L. innocua*, responder populations A and B, and magnetic nanoparticles for self-assembly with responders in three settings: brightfield, distributed (culture before magnet application), and focused (during magnet application).

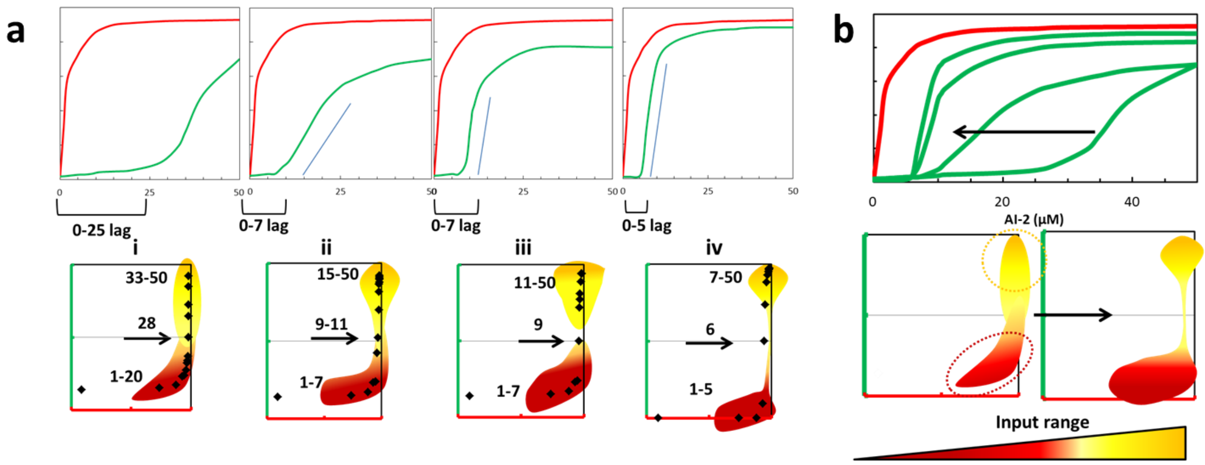

Supplementary Figure 15 Hypothetical response curves to an input range, leading to red and green output. (a) Combinations of response curve pairs, and visual planes of the appearance of red (x-axis) versus green (y-axis) output levels. Input values are defined across the plane. (b) The trend of changing a green response curve with respect to red is summarized. A heat map representation of the data indicates the shift from low to high input concentrations.

Supplementary Table 1 List of strains and plasmids.

Strain/plasmid	Relevant genotype and property	Source or reference
Strains		
E. coli		
W3110	K12 strain, wild type, *λ*-, F-, IN(*rrnD-rrnE*)1, *rph-1*s	Genetic Stock Center Yale University, New Haven, CT
MDAI2	W3110 *luxS*::Tcr W3110-derived *luxS* mutant strain	1
CT104	W3110 *luxS*::Tcr, lsrFG^{-}, W3110-derived *luxS*, *lsrFG* mutant strain	2
BL21 (DE3)	B strain, F– *ompT hsdSB*(rB- mB-) *gal dcm rne131*λ(DE3)	Invitrogen
V. harveyi		
BB170	BB120 *luxN*::Tn5 (sensor 1- ,sensor 2+), Km^{r}	3
L. innocua		4
Plasmids		
pCT6	pFZY1 derivative, containing *lsrR* and *lsrR* promoter region fused with *T7RPol*, Ap^{r}	5
pFZY1	*galK'-lacZYA* transcriptional fusion vector, Ap^{r}	6, 7
pET200/	Cloning vector, containing *T7* promoter, Km^{r}	Invitrogen
pAIDA-I	pACYC184 derivative, containing AIDAc translocation gene, Cm^{r}	8
pSBP-Venus	pET200 derivative, containing *sbp-venus*, AIDAc translocation gene, Km^{r}	This study
pSBP-mCherry	pET200 derivative, containing *sbp-mcherry*, AIDAc translocation gene, Km^{r}	This study
pSBP-eGFP	pET200 derivative, containing *sbp-egfp*, AIDAc translocation gene, Km^{r}	This study

Supplementary Table 2 List of primers for pSBP plasmid constructs.

Oligo name	Sequence (5'—3')*
SBP-mCherry-F	CGGGCGAACTGGAACAGCTGCGCGCGCGCCTGGAACATCATCCGCAGGGCCAGCGCGAACCGggaattaac ATGGTGAGCAAGGGCGAGG
mCherry-XbaI-R	gcgcgcgctctagaCTTGTACAGCTCGTCCATGCCGCC
SalI-SBP-F	gtgtgtgtgtcgacATGGATGAAAAAACCACCGGCTGGCGCGGCGGCCATGTGGTGGAAGGCCTGG CGGGCGAACTGGAACAGCTGCGCG
NdeI-AIDAc- F	cccctctagaaataattttgtttaactttaagaaggagatata catATGAATAAGGCCTACAGTATCATTTGGAGCCACTCCAG
AIDAc-SacI-R	gttagcagccgatcgttgagctcgcccttTCAGAAGCTGTATTTTATCCCCAGTGCTCCGG
SBP-XhoI-Venus-F	GCGAACTGGAACAGCTGCGCGCGCGCCTGGAACATCATCCGCAGGGCCAGCGCGAACCG ctcgagATGAGCAAAGGCGAAGAACTGTTCACGGG
Venus-KpnI-R	cgcgcgcgggtaccTTTATACAGTTCATCCATACCATGGCGTAATGC
Venus-KpnI-Linker-F	GGCCGGCATTACGCATGGTATGGATGAACTGTATAAAggtacc GTGAATAACAATGGAAGCATTGTCATTAATAACAGCATTATAAACGGG
Linker-NotI-R	gcgcgcgcgcggccgcACTTTCTTTTGTAGGATTAAGAATGATATTTTTGTTATTAAC
XhoI-EGFP-F	ctctctctctcgagATGGTGAGCAAGGGCGAGGAGC
EGFP-KpnI-R	agagagagggtaccCTTGTACAGCTCGTCCATGCCGAGAGTGATC

*Capitalized letters indicate complementarity to a gene. Blocked letters indicate a restriction endonuclease site. Underlines indicate complementarity to another primer.

Supplementary Table 3 Template gene sequences inserted on pET200 vectors.

Gene	Sequence
**Signal peptide**	ATGATAAGGCCTACAGTATCATTTGGAGCCACTCCAGACAGGCCTGGATTGTGGCCTCAGAGTT<br>AGCCAGAGGACATGGTTTTGTCCTTGCAAAAAATACACTGCTGGTATTGGCGGTTGTTTCCACA<br>ATCGGAAATGCATTTGCAG
**Streptavidin binding peptide**	ATGGATGAAAAAACCACCGGCTGGCGCGGCGGCCATGTGGTGGAAGGCCTGG**CGGGCGAACTGG**<br>**AACAGCTGCGCG**CGCGCCTGGAACATCATCCGCAGGGCCAGCGCGAACCG
**Venus**	ATGAGCAAAGGCGAAGAACTGTTCACGGGTGTGGTTCCGATCCTGGTTGAACTGGATGGCGATG<br>TGAACGGTCATAAATTTAGCGTGTCTGGTGAAGGCGAAGGTGATGCGACCTACGGCAAACTGAC<br>GCTGAAACTGATTTGCACCACGGGTAAACTGCCGGTTCCGTGGCCGACCCTGGTGACCACGCTG<br>GGTTATGGTCTGATGTGTTTCGCACGTTACCCGGATCACATGAAACGCCATGATTTCTTTAAAT<br>CTGCGATGCCGGAAGGCTATGTGCAGGAACGTACCATCTTTTTCAAAGATGATGGTAACTACAA<br>AACCCGCGCGGAAGTTAAATTTGAAGGCGATACGCTGGTGAACCGTATTGAACTGAAAGGTATC<br>GATTTCAAAGAAGATGGCAATATTCTGGGTCACAAACTGGAATACAACTACAACAGTCATAACG<br>TGTACATTACCGCCGATAAACAGAAAAACGGTATCAAAGCAAACTTCAAAATCCGTCACAACAT<br>CGAAGATGGCGGTGTTCAGCTGGCCGATCATTACCAGCAGAACACCCCGATTGGCGATGGTCCG<br>GTGCTGCTGCCGGATAATCATTATCTGAGTTACCAGAGCAAACTGTCTAAAGATCCGAATGAAA<br>AACGCGATCACATGGTTCTGCTGGAATTTGTGACCGCGGCCGGCATTACGCATGGTATGGATGA<br>ACTGTATAAA
**mCherry**	ATGGTGAGCAAGGGCGAGGAGGATAACATGGCCATCATCAAGGAGTTCATGCGCTTCAAGGTGC<br>ACATGGAGGGCTCCGTGAACGGCCACGAGTTCGAGATCGAGGGCGAGGGCGAGGGCCGCCCCTA<br>CGAGGGCACCCAGACCGCCAAGCTGAAGGTGACCAAGGGTGGCCCCCTGCCCTTCGCCTGGGAC<br>ATCCTGTCCCCTCAGTTCATGTACGGCTCCAAGGCCTACGTGAAGCACCCCGCCGACATCCCCG<br>ACTACTTGAAGCTGTCCTTCCCCGAGGGCTTCAAGTGGGAGCGCGTGATGAACTTCGAGGACGG<br>CGGCGTGGTGACCGTGACCCAGGACTCCTCCCTGCAGGACGGCGAGTTCATCTACAAGGTGAAG<br>CTGCGCGGCACCAACTTCCCCTCCGACGGCCCCGTAATGCAGAAGAAGACCATGGGCTGGGAGG<br>CCTCCTCCGAGCGGATGTACCCCGAGGACGGCGCCCTGAAGGGCGAGATCAAGCAGAGGCTGAA<br>GCTGAAGGACGGCGGCCACTACGACGCTGAGGTCAAGACCACCTACAAGGCCAAGAAGCCCGTG<br>CAGCTGCCCGGCGCCTACAACGTCAACATCAAGTTGGACATCACCTCCCACAACGAGGACTACA<br>CCATCGTGGAACAGTACGAACGCGCCGAGGGCCGCCACTCCACCGGCGGCATGGACGAGCTGTA<br>CAAG
**eGFP**	ATGGTGAGCAAGGGCGAGGAGCTGTTCACCGGGGTGGTGCCCATCCTGGTCGAGCTGGACGGCG<br>ACGTAAACGGCCACAAGTTCAGCGTGTCCGGCGAGGGCGAGGGCGATGCCACCTACGGCAAGCT<br>GACCCTGAAGTTCATCTGCACCACCGGCAAGCTGCCCGTGCCCTGGCCCACCCTCGTGACCACC<br>CTGACCTACGGCGTGCAGTGCTTCAGCCGCTACCCCGACCACATGAAGCAGCACGACTTCTTCA<br>AGTCCGCCATGCCCGAAGGCTACGTCCAGGAGCGCACCATCTTCTTCAAGGACGACGGCAACTA<br>CAAGACCCGCGCCGAGGTGAAGTTCGAGGGCGACACCCTGGTGAACCGCATCGAGCTGAAGGGC<br>ATCGACTTCAAGGAGGACGGCAACATCCTGGGGCACAAGCTGGAGTACAACTACAACAGCCACA<br>ACGTCTATATCATGGCCGACAAGCAGAAGAACGGCATCAAGGTGAACTTCAAGATCCGCCACAA<br>CATCGAGGACGGCAGCGTGCAGCTCGCCGACCACTACCAGCAGAACACCCCCATCGGCGACGGC<br>CCCGTGCTGCTGCCCGACAACCACTACCTGAGCACCCAGTCCGCCCTGAGCAAAGACCCCAACG<br>AGAAGCGCGATCACATGGTCCTGCTGGAGTTCGTGACCGCCGCCGGGATCACTCTCGGCATGGA<br>CGAGCTGTACAAG

**Linker**	GTGAATAACAATGGAAGCATTGTCATTAATAACAGCATTATAAACGGGAATATTACGAATGATG<br>CTGACTTAAGTTTTGGTACAGCAAAGCTGCTCTCTGCTACAGTGAATGGTAGTCTTGTTAATAA<br>CAAAAATATCATTCTTAATCCTACAAAAGAAAGT
**AIDAc**	ATGTATAGGTAATACTCTTACCGTGTCAAATTATACTGGGACACCGGGAAGTGTTATTTCTCTT<br>GGTGGTGTGCTTGAAGGAGATAATTCACTTACGGACCGTCTGGTGGTGAAAGGTAATACCTCTG<br>GTCAAAGTGACATCGTTTATGTCAATGAAGATGGCAGTGGTGGTCAGACGAGAGATGGTATTAA<br>TATTATTTCTGTAGAGGGAAATTCTGATGCAGAATTCTCTCTGAAGAACCGCGTAGTTGCCGGA<br>GCTTATGATTACACACTGCAGAAAGGAAACGAGAGTGGGACAGATAATAAGGGATGGTATTTAA<br>CCAGTCATCTTCCCACATCTGATACCCGGCAATACAGACCGGAGAACGGAAGTTATGCTACCAA<br>TATGGCACTGGCTAACTCACTGTTCCTCATGGATTTGAATGAGCGTAAGCAATTCAGGGCCATG<br>AGTGATAATACACAGCCTGAGTCTGCATCCGTGTGGATGAAGATCACTGGAGGAATAAGCTCTG<br>GTAAGCTGAATGACGGGCAAAATAAAACAACAACCAATCAGTTTATCAATCAGCTCGGGGGGGA<br>TATTTATAAATTCCATGCTGAACAACTGGGTGATTTTACCTTAGGGATTATGGGAGGATACGCG<br>AATGCAAAAGGTAAAACGATAAATTACACGAGCAACAAAGCTGCCAGAAACACACTGGATGGTT<br>ATTCTGTCGGGGTATACGGTACGTGGTATCAGAATGGGGAAAATGCAACAGGGCTCTTTGCTGA<br>AACTTGGATGCAATATAACTGGTTTAATGCATCAGTGAAAGGTGACGGACTGGAAGAAGAAAAA<br>TATAATCTGAATGGTTTAACCGCTTCTGCAGGTGGGGGATATAACCTGAATGTGCACACATGGA<br>CATCACCTGAAGGAATAACAGGTGAATTCTGGTTACAGCCTCATTTGCAGGCTGTCTGGATGGG<br>GGTTACACCGGATACACATCAGGAGGATAACGGAACGGTGGTGCAGGGAGCAGGGAAAAATAAT<br>ATTCAGACAAAAGCAGGTATTCGTGCATCCTGGAAGGTGAAAAGCACCCTGGATAAGGATACCG<br>GGCGGAGGTTCCGTCCGTATATAGAGGCAAACTGGATCCATAACACTCATGAATTTGGTGTTAA<br>AATGAGTGATGACAGCCAGTTGTTGTCAGGTAGCCGAAATCAGGGAGAGATAAAGACAGGTATT<br>GAAGGGGTGATTACTCAAAACTTGTCAGTGAATGGCGGAGTCGCATATCAGGCAGGAGGTCACG<br>GGAGCAATGCCATCTCCGGAGCACTGGGGATAAAATACAGCTTC

Supplementary Table 4 Equations representing fluorescent responder percentages as a function of autoinducer-2 concentration

	**Responder A**	**Responder B**
Equation<br>*f(x) = %([A])*<br>*%*: % expressers<br>*[A]*: AI-2 (µM)	$\% = \frac{\%_{\mathrm{max}}([A])}{\mathrm{K_m} + [A]}$	$\% = \frac{\%_{\mathrm{max}}([A] - C_{lag})}{\mathrm{K_m} + [A] - 2C_{lag}}$
$\%_{\mathrm{max1}}$	100	100
$\%_{\mathrm{max2}}$	50	50
$\mathrm{K_m}$	1.99	20.86
C_{lag}	--	5

Supplementary Table 5 Equations for parallel populations that generate red and green response patterns with input *x*.

Red $f(x)$	Green $f(x)$	
$\boldsymbol{\frac{x^2}{5} + \frac{x}{20}}$	$\boldsymbol{\frac{x^2}{10} + \frac{1}{2}}$	(1)
$5x + 50$	$5x - 40$	(2)
$10x$	$\frac{400x}{x + 10}$	(3)
$5x + 50$	$x + 10$	(4)
$\frac{1000x}{x + 30}$	$5x + 10$	(5)
$\frac{x^3 + x^2 + 200}{400}$	$\frac{600x}{x + 3}$	(6)
$\frac{300x}{2 + x}$	$\frac{200x}{20 + x}$	(7)
$\frac{x^2 + 100x + 30}{5}$	$2x + 2$	(8)
$x + 2$	$\frac{x^2 + 50x + 30}{5}$	(9)
$100 - 100\sin(\frac{x}{2})$	$2x$	(10)

Supplementary Methods

Vector Construction

A series of cloning steps were performed to generate three plasmids that utilize the AIDAc membrane transporter for the surface display of SBP-tagged fluorescent proteins: pSBP-mCherry, pSBP-Venus, and pSBP-eGFP (Supplementary Table 1). Oligomers in Supplementary Table 2 were used , initially, for gene fusion within pAIDA-I, with template gene sequences listed in Supplementary Table 3. First, one-pot nested PCR was performed using *mcherry* as a template and 0.2 μM SBP-mCherry-F and 0.5 μM mCherry-XbaI-R as oligomers. After 5 cycles, a minimal volume of 100 μM SalI-SBP-F was added to achieve 0.5 μM in the working PCR reaction to further extend the 5' end for another 30 cycles. The vector pAIDA-I and the PCR-generated sequence were double-digested with SalI and XbaI in order to insert *sbp-mcherry* as the passenger in the AIDAc construct. Next, NdeI-AIDAc-F and AIDAc-SacI-R were used as primers to PCR the gene fusion of *signal peptide-sbp-mcherry-AIDAc* from start to stop codon. The destination vector, pET200, and the PCR product were each digested with NdeI and SacI to insert the gene fusion after a T7 promoter. The resulting plasmid was named pSBP-mCherry.

Additional measures were taken to allow for more facile cloning of a variety of fluorescence genes. The one-pot nested PCR strategy was used to amplify *sbp-venus* using SBP-XhoI-Venus-F, SalI-SBP-F, and Venus-KpnI-R as primers, introducing new restriction sites directly up and downstream of *venus*. A linker upstream of *AIDAc* was also PCRed using primers Venus-KpnI-Linker-F and Linker-NotI-R, which extended the sequence to include an overlap region with *venus*. Next, the two PCR products, *sbp-venus* and the linker, were annealed together in a PCR reaction to link the two sequences together and amplify their combination; no primers were necessary since the sequences shared complementarity at the 3' end of *venus*. The PCR product

was digested along with pSBP-mCherry using SalI and NotI to replace *mcherry* upstream of *AIDAc*. The completed construct was named pSBP-Venus.

Finally, XhoI-eGFP-F and eGFP-KpnI-R were used as primers to amplify *egfp*. Digestion of both *egfp* and pSBP-Venus with XhoI and KpnI were followed by routine insertion to swap *venus* for *egfp*. The resulting plasmid was named pSBP-eGFP. Constructs for each engineered plasmid are shown in Supplementary Fig. 1.

Microscopy visualization of cell-nanoparticle interactions

Interactions between cells expressing SBP and streptavidin-conjugated magnetic nanoparticles (mNP, 100 nm dia., Chemicell) were investigated. In Supplementary Fig. 3a-b, scanning electron micrograph images of each species ((a) cell and (b) mNPs) were used as controls for interpretation of subsequent SEM images showing combinations of the two species. In Supplementary Fig. 3b, it is observed that the mNPs frequently exist in clusters of various sizes, some of which have dimensions smaller than a cell (~ 1 x 2.5 μm, Supplementary Fig. 3a) and some of which are greater. It was not clear whether SEM sample preparation contributed to the clustering effect.

To investigate further, fluorescence labeling was applied for FACS and fluorescence microscopy. Supplementary Fig. 3c shows a scheme of the fluorescence designation and assembly of components. Cells expressing SBP-mCherry (or uninduced for expression as a negative control) were fixed with 2 % paraformaldehyde, washed in 10 mM PBS, and stained with 13 μM propidium iodide (PI, Life Technologies); thus red cellular fluorescence was due to both mCherry and PI. Next, mNPs were diluted 50-fold from stock into the cell samples. Subsequently, FITC-labeled polyclonal anti-streptavidin IgG (Abcam, #ab7238), diluted 200-

fold in PBS, was incubated with the cell-mNP hybrids for 30 min on ice. Finally, the samples were either imaged directly (Supplementary Fig. 3e) or treated with a magnet on ice; the supernatant was separated from the magnetically-recovered pellet, which was resuspended in an equal volume of PBS and remagnetized three times to filter out cells not bound to mNPs.

FACS was used to look at the distribution of red and green fluorescence in the cell population, but to also count the magnetically recovered fractions of controls (+/- SBP-mCherry expression) in fixed volumes. Supplementary Fig. 3d shows the counted density of cells, collected in triplicate from controls of equal volume. Cells were found in the negative control sample, indicating some extent of non-specific interactions with mNPs. However, an approximately three-fold greater cell density was recovered in the positive control. T-test analysis indicated that the results are statistically significant with $p < 0.05$. Thus, mNPs perform favorably for selecting SBP-expressing cells compared to non-expressing cells. Furthermore, as shown by the inset FACS plot, a large portion of the magnetically recovered cells exhibited strong fluorescence at both red and green wavelengths, verifying that an abundance of the recovered cells were bound to FITC-labeled mNPs.

Lastly, confocal microscopy was used to visualize cell-mNP complexes. In Supplementary Fig. 3, samples were compared before (e) and after (f) refining mNP-bound cells within a magnetic field. In this way, the samples were examined for an effect of clustering as a consequence of exposure to a magnetic field. Supplementary Fig. 3e shows a collection of cells, of which some are unbound and some are bound to green mNPs; of the bound cells, two appear to interact closely with a large mNP cluster (~ 2 μm in diameter) and one cell appears to have at least three single particles bound to the cell surface (highlighted by an arrow). The particle diameters

appear to be greater than 100 nm, which could either be due to a larger-than-average size or due to a fluorescence halo-effect.

By contrast, in Supplementary Fig. 3f, a large multicellular and multiparticular conglomerate is observed. Many cells appear to be irreversibly embedded within the mass and many particle clusters appear to multivalently bind several cells. This massive clustering (characteristically larger than the size of a single cell) was only observed post-magnetic focusing, and is likely due to the strong magnetic force pulling all species to the same destination (magnet's edge) and their consequential confinement there before resuspension. It is reasonable to expect mNPs to bind with multivalency since streptavidin is multivalent (tetrameric with four binding sites) and a single mNP should be conjugated with several streptavidin molecules. Thus, the amalgamation observed in Supplementary Fig. 3f is likely to occur at a magnet's surface upon redistributing species (from dispersed to focused) within the magnetic field.

Western blot of membrane-expressed SBP-fluorophore-AIDAc

Figure 4 shows western blot images for analysis of the size, location (cytoplasm or membrane), and relative expression level of SBP-fluorophore fusions. All combinations of the SBP-tagged fusion protein, containing SBP, either Venus, mCherry, or eGFP, a linker, and AIDAc were predicted to be 88 kDa in size. In the literature, AIDAc has been reported at 51 kDa in size; when fused with a passenger, the resolved size of the fusion is often smaller than its prediction[8]. In Figure 5a, Type A and B cells with pCT6 and either pSBP-Venus, pSBP-mCherry, or pSBP-eGFP were compared for expression. On the western blot, no SBP-tagged protein was detected in rows to the left of the ladder, which were loaded with the cytosoplasmic protein fractions. Heavy bands are observed to the right of the ladder, containing the membrane fractions. The stark difference in SBP-tagged protein composition between lysate fractions indicates that nearly

all of the AIDAc-fused passenger is located on/in membrane rather than cytoplasm. Secondly, in the lanes loaded from the lysate pellet, protein from Type A cells, regardless of the fluorophore variant, show a stronger signal than Type B. This indicates that protein the expression level induced by 28μM AI-2 is higher in Type A cells. Finally, the blots show evidence that the SBP fusion is expressed with different efficiency depending on the fluorescent protein inserted. The band corresponding to each SBP-fluorophore-AIDAc fusion appears near 82 kDa. At this band, fusion proteins containing Venus and eGFP are expressed at higher levels than mCherry, in both A and B cell types. The corresponding SDS-PAGE gel shows nearly the same amount of protein per well, confirming that differences in band intensity on the blot are due to expression rather than a discrepancy in protein loading.

Type A and B cells were compared for SBP-Venus-AIDAc expression over time after induction with 20 μM AI-2. The western blots in Figure 5b-c resolve the (b) membrane and (c) cytoplasmic fractions for samples isolated at time intervals after induction (time 0). In Figure 5b, a band cluster develops over time at 82 kDa for both Type A and B cells, with a stronger signal from A expression. For both Type A and B cells, 3 h is required before the band intensity reaches above-background levels, from which can be inferred as the approximate time requirement for protein targeting to the membrane, maturation, and translocation. Strong expression is maintained from 3 – 7 h, decreasing slightly by 8 h and further diminished after overnight culturing, likely due to slight degradation. The 82 kDa signal is observed as a tight cluster of bands, with the largest two appearing as a pair of distinct bands, and a smear below. While it is not clear whether all constituents represent the full sequence of SBP-Venus-AIDAc, we hypothesize that the paired bands could be identified as the full fusion before (upper) and after (lower) cleavage of the signal peptide, reducing the size by approximately 5 kDa. At low

expression levels, cleavage could occur in all expressed protein, resulting in only the lower band and slight emergence of the upper band in some cases (Type A: 0, 2 h; Type B: all timepoints). Once overexpressed, a percentage of the fusion protein remains uncleaved (other timepoints). The lower smear could be explained by growing protein-in-translation, which is reduced over time (by 24 h) and at lower expression levels (Type B cells).

We note that in Figure 5c (the cytosolic fractions), no bands at 82 kDA are observed at any timepoint and in either cell type. This absence indicates that at the onset of expression, the protein is immediately targeted to the membrane- evidence of highly efficient surface display.

Superficial immunoprobing of AIDAc-linked eGFP

Having probed the engineered cell surfaces for the availability of SBP, we also investigated whether the fluorescent protein segment of the fusion protein also translocated across the outer membrane. Specifically, we probed cell surfaces for eGFP using an anti-eGFP antibody. Immunoprobing was performed on cells that had been treated to express SBP-eGFP-AIDAc and on a negative control that did not express the fusion protein. First, cells were washed in 10 mM PBS and incubated with anti-GFP monoclonal mouse IgG (Rockland Immunochemicals, #600-301-215), diluted 1:100 in PBS, for 1 h at room temperature. Samples were washed twice in PBS, then incubated with Alexafluor594 (red) anti-mouse polyclonal goat IgG (Molecular Probes, #A-11032) as a secondary antibody, diluted 1:200 in PBS, again for 1 h at room temperature. A sample of cells expressing eGFP was not treated with the secondary antibody to act as a negative control in calibrating the fluorescence signal contributed by the red probe. Figure 6 shows a scheme of the conditions tested and results from flow cytometry and microscopy.

FACS was used to analyze cells for green (eGFP) and red (immunostaining) fluorescence per cell and the intensities of each fluorophore were plotted as coordinates, with the fluorescence map divided into quadrants: Q1, significant red fluorescence, Q2, cells exhibiting both red and green, Q3, non-fluorescing cells, and Q4, green only. In Fig. 6a, immunostained cells expressing eGFP showed strong green fluorescence, mapping in Q4, with a distinct subset mapping to Q2 for cells colocalized with red and green. By contrast, non-immunostained eGFP cells are negligible in Q2, appearing, instead in Q4 (Fig. 6b), and cells that do not express eGFP appear mostly in Q3 due to the absence of both fluorophores (Fig. 6c).

This data was visually corroborated by microscopy. Low magnification shows a large sample size of fluorescent cells, with the boxed areas split into green and red channels. Additionally, higher magnification was used to visualize the cell surface localization of the fluorescent components. Interestingly, fluorescence appears in clusters, around the perimeter of the cell cross-section. Only cells in Fig. 6a shows both green and red fluorescence. The positions on the cell surface where eGFP is expressed are identically colocalized with red; this evidence of positive cell-surface immunoprobing suggests that when SBP-eGFP-AIDAc is expressed, the entire SBP-eGFP fusion translocates across the outer membrane for many cells, shown by Fig. 6a. While not explicitly tested, we hypothesize that the other tagged fluorescent proteins may translocate as well, especially considering that the SBP portion was at least confirmed to be surface-accessible in all variants (Fig. 2).

Autoinducer-2 bioassay

Autoinducer-2 was analyzed by a standard bioassay where bioluminescence intensity corresponds to relative AI-2 concentration.[9] Specifically, *Vibrio harveyi* strain BB170 generates bioluminescence in response to AI-2, and provides a quantifiable measure of extracellular AI-2

level. A standard assay was performed by diluting a BB170 16 h culture 1:5000 into fresh AB medium. Diluted BB170 (180 μL) was mixed with each sample (20 μL) and incubated in 5 mL culture tubes for 4.5 h at 30 °C, 250 rpm shaking, then recording the bioluminescence measurement from each sample. Luminescence measurements were obtained using a luminometer (EG&G Berthold, Gaithersburg, MD) and are reported as a fold change compared to a blank LB sample. To study the dynamics of AI-2 production by W3110, conditioned media were obtained from W3110 during growth in LB medium at 30 °C, 250 rpm shaking. The culture sample was pelleted and supernatant media was filter-sterilized. AI-2 activity from W3110 throughout growth is reported in Supplementary Fig. 7, normalized to a sample assayed with blank LB medium.

AI-2 responder coculture inoculated with cell types A and B

First, we evaluated the robustness of the populations' fluorescence response as accurate AI-2 output. From the data in Fig. 5b of the main text, response patterns follow a Michaelis-Menten or Monod-type relationship.   Thus, equations were established to mathematically characterize the percent of SBP-fluorescence expressing cells as a function of AI-2 concentration, using an empirically-defined Monod constant and adjusted maximum percentage for each population type (Supplementary Table 4). The correlation of the FACS data and population models (with the maximum possible % set at 100) are shown in Figure 8a; the general relationship between AI-2 and the fluorescently responding population is satisfied with R^2 correlations greater than 0.87.

To understand the necessary coculture-adjustments for the models, AI-2 responder strains A and B were evaluated for growth as a consequence of coculturing. First, Type A cells + pSBP-mCherry and Type B + pSBP-eGFP were cultured separately until mid-exponential phase, then induced with 25 μM AI-2. The growth rates, measured as the optical density at 600 nm

(OD600), of A and B once induced were compared between single cultures and a coculture comprising a 1:1 volume ratio of each strain. Supplementary Fig. 8c shows the OD600 trends for the cultures approximately 5 h post-induction. From this data, correlated growth of each culture type is observed throughout exponential (0 to 3.5 h) and stationary phase. Since the growth rates of Type A and B match, equal growth can be surmised in a coculture scenario, where the total population composition is approximately 50 % each of the two cell types throughout incubation. Accordingly, the coculture growth data matches that of both single cultures, indicating that coculturing causes no change in growth dynamics for either cell type.

The models of A and B populations were used to evaluate our A and B expression data in coculture as a function of AI-2 (obtained from Fig. 5d). Based on Supplementary Fig. 8c, we hypothesized that to fit the model in a coculture scenario, $\%_{max}$ becomes reduced to 50 (out of 100) to satisfy the strain's composition within the master population, but the response function to AI-2 should remain otherwise independent of coculturing. Supplementary Figure 8b plots red A and green B data as raw numbers (data from Fig. 5d in the main text, diluted ten-fold) and the coculture-adjusted models as cell number (per 100 cells in the total population). General fits between data and models are observed for both cell types. These comparisons suggest that response to AI-2 from A and B populations is independent of coculturing, but that the muted fluorescent output, as a % of the population, is due to a lower $\%_{max}$ during coculture.

Finally, to fully evaluate the established behavior models for Type A and B cells in coculture, we considered the master population response, or in other words, the sum of A and B output, plotted in Supplementary Fig. 8d. Additionally, the resolution of A, B, and the population as a whole, was considered across the range of AI-2, plotted as the change in response per μM AI-2 (Supplementary Fig. 8d). From this analysis, the master population takes on properties of A and

B at different subsidiary ranges. At low concentrations (< 5 μM), the master population response is primarily due to A turning on. The response in this range has a steep slope, giving both A and A+B a lower detection limit and higher resolution than B alone. However, the slope of the A quickly diminishes as the A-contributed response plateaus at the same range in which B contributes a dynamic response to AI-2 (> 5 μM). At this range, the total responding population is contributed by a steep accumulation of Type B and a more gradual accumulation of A cells, making the overall resolution higher than that of either A or B alone; moreover, the responsive range of the total population is expanded beyond that of either constituent. Thus, coculturing two responders in parallel enhances the population-based AI-2 sensing while simultaneously generating a color-accumulation index (first, red A and second, green B).

Image analysis-based identification of strains in coculture

Pixel-based particle analysis performed with ImageJ was used to quantify the unique size distributions of each cell type: *L. innocua* (Syto9-stained AI-2 producer) is definitively smaller than SBP-Venus-expressing BL21(DE3) (Supplementary Fig. 9). Furthermore, fluorescence imaging was used to observe differential green and red signals through each respective filter. The Syto9-stained producer exhibits stronger green fluorescence than red, while the signal from the Venus-expressing cell is composed of nearly equal green and red intensities (Supplementary Fig. 10).   The size and fluorescence features of each cell type were used to quantify cell numbers for further image analyses.

Cell sorting using streptavidin-coated magnetic nanoparticles

Magnetic activated cell sorting. Culture samples were first washed by resuspension in cold PBS. Initially, *L. innocua* was fluorescently stained with Syto9 nucleic acid stain (Life Technologies) according to protocol. Cell densities were readjusted to an OD of 0.2 and then diluted 10-fold in

PBS. Mixed cultures were prepared using a 1:1 volumetric ratio of each cell type. Streptavidin-conjugated magnetic nanoparticles (mNPs, 100 nm diameter) were first washed in cold PBS, then diluted from stock concentration approximately 20-fold into cell samples, creating a 50,000-fold particle-to-cell excess, similar to previously published methods.[11]

After incubating the cells and mNPs in a 96-well plate at 4 °C for 20 min, a 2.2 x 1.6 cm (dia. x ht.) neodymium N42 magnet (K and J Magnetics) was placed under each well for 3 min to collect mNPs on the bottom surface. The supernatant was removed and particles were washed twice by resuspending in the same volume of cold PBS and magnetically recollecting; the first supernatant, reproducibly containing the vast majority of unbound cells, is referred to as the eliminated fraction. The recovered sample, consisting of the magnetically collected pellet, was resuspended in the same PBS volume. Fractions were counted by imaging samples at 200x magnification. To image magnetic collection *in situ*, a 1.6 x 0.8 mm (dia. x ht.) N52 neodymium magnet was taped behind a coverslip. A 2 μL mixture of cells and mNPs was added to the opposite side of the coverslip, directly on top of the magnet, set for 2 min, after which another coverslip sealed the sample for imaging.

Focusing magnetically-equipped processor cells.

We evaluated the application of magnetic collation as a form of abiotic processing in order to focus magnetically-equipped cell units into aggregate multi-unit fluorescence data. The ability to selectively reposition "on" cells only was incorporated by design: since SBP is fused to the fluorophore terminus, SBP-surface exposure is conditional to fluorescence output, as is cell hybridization with streptavidin-conjugated mNPs (Supplementary Fig. 11a). In this way, a

magnetic field should filter AI-2 producers and off cells from a diverse distribution and simultaneously focus mNP-attached cells to the magnetic source.

We used induced cells with expressed SBP-Venus as the processing unit and *L. innocua* as the AI-2 producer. The AI-2 responder emitted fluorescence due to Venus and we used a Syto9 nucleic acid stain to make the AI-2 producer also fluoresce. It was verified that the cell types could be tracked by fluorescence microscopy and distinguished from one another. Image processing-based tracking of the two cell types is shown by Supplementary Figs. 9 and 10. We performed experiments where the cells were mixed with mNPs to briefly allow hybridization, then a magnet was applied. This process formed two fractions: a focused fraction (magnetically recovered cells), contents concentrated at the magnet surface, and a filtered fraction (eliminated cells), which comprised unbound cells remaining in the supernatant and separated from the magnetized portion (Supplementary Fig. 11a).

First, we directly observed the magnetic behavior of cell-mNP complexes by imaging the samples *in situ* with a magnet. A 1.6 mm diameter neodymium disc was fixed beneath a coverslip; subsequently the cell-mNP mixture was added to the coverslip and a composite image was taken to include directly adjacent fields of view. Supplementary Fig. 11b shows the resulting image, with merged brightfield and fluorescence views, overlaid with a plot where the number of cells were counted as a function of position across the field of view (x position). The magnet is visible as the dark section on the left-hand side of the image. At the location of the magnet, the recovered cell density is observably higher, by a count of at least 100-fold, than the eliminated cell density at a distance from its edge; the sharp drop in cell count occurs within 1 mm of the magnet's edge. Contrasted against a diffuse distribution of processor cells, each transmitting a single optical datum, collective fluorescence output from the focused population is

significantly sharper and more obvious. Thus, magnetic enrichment is effective at focusing the individual fluorescence signals due to the physical act of concentration.

To quantitatively characterize the efficacy of magnetic parsing, cells in sample fractions were counted from images taken before (pre-) and after magnetic parsing, at which point fractions were split between eliminated (E) and recovered (R). Magnetic redistribution for processor cells, AI-2 producers, and a mixture of the two were completed in triplicate. Results show consistency magnetic filtering across samples (Supplementary Fig. 11), where each sample underwent two sequential separations. Venus-expressing processor cells were recovered at greater than 40 % while less than 30 % were eliminated. Conversely, an average of 80 % of the AI-2 producer, *L. innocua*, was eliminated and less than 5 % was recovered. When mixed together, performance was comparable to that of the unmixed samples. Of the 90 % of cells eliminated, 20 % were the processor cells and over 60 % were *L. innocua*. Nearly 40 % of the cells in the original sample were recovered; composed mostly of processors, with less than 5 % being *L. innocua*.
Thus, magnetic post-processing significantly refined the mNP-equipped SBP-Venus expressers, filtering out the majority of other constituents. The magnetic field also compacted the position of the Venus-expressing majority, thereby concentrating the fluorescence signal into collective output (Supplementary Fig. 11). These data verified the specificity of MACS for SBP-expressers over *L. innocua* and provided evidence that self-assembled mNP-cell complexes are capable of selective positioning, prompted by the stimulus of a magnetic field.

Compared to the results described by Supplementary Fig. 3, where SBP-dependent specificity was evaluated, results in Supplementary Fig. 11c show improved specificity, although non-fluorescing non-expressers were excluded. Supplementary Fig. 11c instead compares specificity against *L. innocua*, which in addition to lacking SBP, is also Gram-positive and therefore has a

distinctly different cell wall than *E. coli*. It is not clear whether competition for streptavidin binding in mixed cultures, which occurs more favorably for SBP-expressers compared to non-expressers (Supplementary Fig. 3d) and compared to *L. innocua* (Supplementary Fig. 11c), results in interactions with enhanced specificity. Regardless, a key advantage is that since SBP-expressers are favorably recovered, their inclusion with non-specifically-bound cells does not impact the reliability of the fluorescent result; fluorescence output is inherent to positive expression and has been separately shown to be specific to the molecular input with low noise (Supplementary Fig. 4c).

Evaluation of AI-2 producer and responder coculture dynamics

The dynamics of coculturing were elucidated through a combination of tracking the optical density (OD) of cultures at 600 nm, plated colony counts, and gram staining. Single- and co-cultures were inoculated identically with relevant strains. Throughout the initial 3 h of incubation, the coculture OD followed that of *L. innocua*, while growth from A and B responders was negligible (Supplementary Fig. 12a). This indicates that *L. innocua* proliferation was predominant within the coculture, and further, that its growth was unaffected by the presence of *E. coli* cells. After 3h, *L. innocua* reached stationary phase at an approximate OD of 0.9, which is consistent with its reported range[4]. At the same time, the growth of A and B responders became measurable and indicative of their exponential growth (Supplementary Fig. 12a). The OD of the coculture also continued to increase, presumably due to the exponential accumulation of A and B despite stationary *L. innocua* growth.

Supplementary Fig. 12b reports the measured final ODs after 10 h growth and the onset of stationary phase in each culture. Observed, A and B responders reach similar ODs (2.61 and 2.73, respectively), while the growth rate for *L. innocua* does not surpass 0.9. The coculture

reaches an OD of 2.7, the same as the culture density achieved by A and B. The coculture data suggests that because of an early null net growth from *L. innocua*, the *E. coli* constituents were eventually (after 4 h) able to outgrow *L. innocua*, perhaps by scavenging unused nutrients[4]. However, the fraction of *L. innocua* maintained at stationary phase was not clear from this data.

To investigate the final coculture composition further, culture dilutions were plated onto agar mixed with either brain-heart infusion (BHI) medium, which supports the growth of both *L. innocua* and *E. coli*, or Luria broth (LB) with kanamycin and ampicillin (50 μg mL^{-1} each), permitting only colonies from A and B responders. Colony counts revealed a 1.6 coculture ratio between A + B and *L. innocua*, where the responders comprised just under two-thirds of the culture (Supplementary Fig. 12c). This fraction was found to be consistent with the OD analysis, given the postulation that the *L. innocua* density remains steady. Thus, evidence suggests that *L. innocua* competes sufficiently for nutrients during *E. coli* accumulation in coculture.

The coculture was determined to transition from a state of *L. innocua* abundance to one predominated by A and B responders. Two visualize these two prevailing conditions, Gram staining was applied to samples and imaged. *L. innocua*, a Gram-positive strain, stains violet and *E. coli,* Gram-negative, stains pink-red. In Supplementary Fig. 12d-e, a sample isolated at 3 h reveals a majority of *L. innocua* cells while a 10 h sample shows a prevalence of *E. coli* in addition to *L. innocua*. The images, thus, corroborate the coculture data.

Information processing by distribution among *Listeria innocua*

Cocultures were established directly with an AI-2 producer under conditions that biased AI-2 accumulation such that it presided within each responder's resolution range. Having established that type A cells elicit sensitive response at low AI-2 levels and type B during high AI-2

exposure, we explored the ability of each cell type to engage in processing AI-2 information in an environmental scenario. *Listeria innocua* was used as a target AI-2-producing cell type at fixed initial proportions of AI-2 producer to processing unit in varied culturing media.

First, we tested non-ideal growth conditions in order to evaluate the ability of population A to respond to AI-2 while coresiding with *L. innocua* in a growth-minimized scenario. To create a growth-limiting environment, brain-heart infusion (BHI) was mixed with Luria-Bertani broth (LB) and diluted 10-fold in sterile PBS. A test culture showed, through plating and colony counts, that *L. innocua* proliferated by only three generations in these conditions. *L. innocua* was grown to an OD of 0.3, then rinsed and resuspended in the diluted media. The culture was serially diluted 10-fold and 100-fold, then incubated with population A, resulting in producer-to-processor ratios of 2, 20, and 200, from the most dilute to most dense initial conditions.

Alternatively, we used richer media to portray more optimal conditions for growth and proliferation, having essential nutrients readily available. We observed that cocultures with *L. innocua* proliferate by approximately six generations to reach cell densities at least 10^2 higher than the nutrient-limiting conditions (Supplementary Fig. 13, bottom). Consequently, we used these conditions to support high microbial community densities and tested the capability of population B to interpret AI-2 activity. *L. innocua* was adapted to media composed of 25 % BHI in LB and probed at low exponential phase points, throughout which AI-2 accumulation occurs.[4] Population B was distributed directly into the growing *L. innocua* culture to create inoculation ratios with the AI-2 producer between 75 and 600:1 and coincubated.

The FACS analyses in Supplementary Fig. 13 (top graphs) compare the "consensus" percentages post-culturing, or in other words, the cells in which fluorescence has been triggered after

processing available AI-2. The data show that quorum sensing by *L. innocua* is detectable by population A when introduced to the thinnest population (10^6 CFUs, established from a 2:1 ratio with *L. innocua*) (Supplementary Fig. 13a). While Type B was not statistically different from the control in the dilute conditions, an emergence of Venus-expressers is observed in conditions permitting higher cell density; the trend starts at a producer-to-processor ratio of 75:1, with the resulting percentage of "on" units being statistically significant (Supplementary Fig. 13b). Not reported, we observed that the medium composition, particularly BHI, interfered with induction of AI-2 processing. Previously, interference in standard AI-2 detection methods for *L. monocytogenes* has was attributed to glucose content and glucose has also been shown to favor QS repression in *E. coli* QS reporting. [7, 10] Nevertheless, detection of *L. innocua* was possible in each medium here and exhibits the property of reliable performance in dynamic living environments, enabled by the use of many cell "processing units" for population-based information acquisition.

Magnetically refined distribution and composition evaluation

We explored the expression of each cell type while cocultured across a range of AI-2 concentrations. Additionally, streptavidin-magnetic nanoparticles were applied to bind and concentrate all SBP-expressers within a magnetic field. Image analysis was used to calculate the ratio of red Type A to green Type B while distributed in culture and then at the higher density resulting from magnetic focusing; this data is plotted in Supplementary Figure 14a for each AI-2 condition. The analysis indicated that similar ratios between responders were maintained before and after magnetic collation. At all concentrations, the ratio is of the same order of magnitude and drops sharply between 5 and 11 μM AI-2, due to the emergence of green responders. Supplementary Fig. 14b, shows a comparison of red and green output from cocultures with *L. innocua* at either low or high cell densities, both while the AI-2 processing cells are distributed throughout the culture and then after they have been magnetically focused. Visually, a higher fluorescence density is apparent and enables collective interpretation of population feedback.

Supplementary References

1. DeLisa, M. P.; Valdes, J. J.; Bentley, W. E., Mapping stress-induced changes in autoinducer AI-2 production in chemostat-cultivated Escherichia coli K-12. *Journal of Bacteriology* **2001,** *183* (9), 2918-2928.
2. Wu, H. C.; Tsao, C. Y.; Quan, D. N.; Cheng, Y.; Servinsky, M. D.; Carter, K. K.; Jee, K. J.; Terrell, J. L.; Zargar, A.; Rubloff, G. W.; Payne, G. F.; Valdes, J. J.; Bentley, W. E., Autonomous bacterial localization and gene expression based on nearby cell receptor density. *Mol Syst Biol* **2013,** *9*, 636.
3. Bassler, B. L.; Wright, M.; Showalter, R. E.; Silverman, M. R., Intercellular signalling in *Vibrio harveyi*: sequence and function of genes regulating expression of luminescence. *Mol Microbiol* **1993,** *9* (4), 773-86.
4. Yang, L.; Portugal, F.; Bentley, W. E., Conditioned medium from Listeria innocua stimulates emergence from a resting. *Biotechnol Prog* **2006,** *22* (2), 387-93.
5. Tsao, C. Y.; Hooshangi, S.; Wu, H. C.; Valdes, J. J.; Bentley, W. E., Autonomous induction of recombinant proteins by minimally rewiring native quorum. *Metab Eng* **2010,** *12* (3), 291-7.
6. Koop, A. H.; Hartley, M. E.; Bourgeois, S., A low-copy-number vector utilizing beta-galactosidase for the analysis of gene control elements. *Gene* **1987,** *52* (2-3), 245-56.
7. Wang, L.; Hashimoto, Y.; Tsao, C. Y.; Valdes, J. J.; Bentley, W. E., Cyclic AMP (cAMP) and cAMP receptor protein influence both synthesis and uptake of extracellular autoinducer 2 in Escherichia coli. *J Bacteriol* **2005,** *187* (6), 2066-76.
8. Nhan, N. T.; Gonzalez de Valdivia, E.; Gustavsson, M.; Hai, T. N.; Larsson, G., Surface display of Salmonella epitopes in Escherichia coli and Staphylococcus carnosus. *Microb Cell Fact* **2011,** *10*, 22.
9. Surette, M. G.; Bassler, B. L., Quorum sensing in Escherichia coli and Salmonella typhimurium. *Proc Natl Acad Sci U S A* **1998,** *95* (12), 7046-50.
10. Turovskiy, Y.; Chikindas, M. L., Autoinducer-2 bioassay is a qualitative, not quantitative method influenced by glucose. *J Microbiol Methods* **2006,** *66* (3), 497-503.
11. Lee, J. J.; Jeong, K. J.; Hashimoto, M.; Kwon, A. H.; Rwei, A.; Shankarappa, S. A.; Tsui, J. H.; Kohane, D. S., Synthetic ligand-coated magnetic nanoparticles for microfluidic bacterial separation from blood. *Nano Lett* **2014,** *14* (1), 1-5.
